# A post-synthetic modification strategy for enhancing Pt adsorption efficiency in MOF/polymer composites†

**DOI:** 10.1039/d4sc00174e

**Published:** 2024-05-10

**Authors:** Till Schertenleib, Vikram V. Karve, Dragos Stoian, Mehrdad Asgari, Olga Trukhina, Emad Oveisi, Mounir Mensi, Wendy L. Queen

**Affiliations:** a Institute of Chemical Science and Engineering (ISIC), École Polytechnique Fédérale de Lausanne (EPFL) Rue de l'industrie 17 1951 Sion Switzerland wendy.queen@epfl.ch; b Swiss-Norwegian Beamlines, European Synchrotron Research Facilities (ESRF) BP 220 Grenoble France; c Department of Chemical Engineering and Biotechnology, University of Cambridge CB3 0AS Cambridge UK; d Interdisciplinary Center for Electron Microscopy, École Polytechnique Fédérale de Lausanne (EPFL) CH-1015 Lausanne Switzerland

## Abstract

Growing polymers inside porous metal–organic frameworks (MOFs) can allow incoming guests to access the backbone of otherwise non-porous polymers, boosting the number and/or strength of available adsorption sites inside the porous support. In the present work, we have devised a novel post-synthetic modification (PSM) strategy that allows one to graft metal-chelating functionality onto a polymer backbone while inside MOF pores, enhancing the material's ability to recover Pt(iv) from complex liquids. For this, polydopamine (PDA) was first grown inside of a MOF, known as Fe-BTC (or MIL-100 Fe). Next, a small thiol-containing molecule, 2,3-dimercapto-1-propanol (DIP), was grafted to the PDA *via* a Michael addition. After the modification of the PDA, the Pt adsorption capacity and selectivity were greatly enhanced, particularly in the low concentration regime, due to the high affinity of the thiols towards Pt. Moreover, the modified composite was found to be highly selective for precious metals (Pt, Pd, and Au) over common base metals found in electronic waste (*i.e.*, Pb, Cu, Ni, and Zn). X-ray photoelectron spectroscopy (XPS) and *in situ* X-ray absorption spectroscopy (XAS) provided insight into the Pt adsorption/reduction process. Last, the PSM was extended to various thiols to demonstrate the versatility of the chemistry. It is hoped that this work will open pathways for the future design of novel adsorbents that are fine-tuned for the rapid, selective retrieval of high-value and/or critical metals from complex liquids.

## Abstract 2

A post-synthetic modification strategy was developed to graft metal chelating thiols to polydopamine inside Fe-BTC. X-ray absorption spectroscopy revealed interesting redox properties of the composite that help extract and reduce Pt(iv) from liquid.

## Graphical abstract

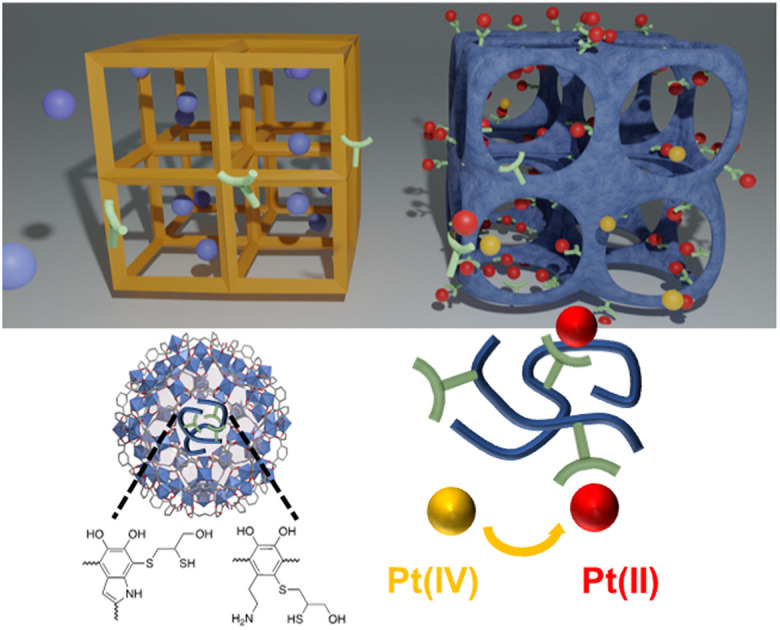

## Introduction

Pt is classified as a critical metal,^1^ in part because it is essential to the impending energy transition.^2^ For instance, it is used as a catalyst in fuel cells^3,4^ and electrolyzers for green hydrogen production,^5^ among other low-carbon technologies. In 2021, mining covered 84% of the global annual Pt demand (208 tons), and the remaining 16% came from recycling.^6^ In view of the forecasted growth in Pt demand and the declining grade of ore in mines around the world,^7,8^ Pt recovery from secondary sources, like end-of-life products, is becoming increasingly important.^1,9^ In contrast to traditional high temperature pyrometallurgical approaches, hydrometallurgy is considered to be more environmentally benign, less energy intensive, and more adaptable at small scales, as needed for dispersed waste sources, like electronic waste.^10^ The method entails leaching metals from solid waste into aqueous media followed by selective metal recovery using a number of different approaches. While adsorption has emerged as one of the most viable and sustainable approaches to recover targeted metal from liquids,^10^ many existing adsorbents lack adequate capacity or selectivity, particularly when the desired metal is found in low concentrations and highly complex environments (with many other competing species). To overcome this, research efforts are aimed at decorated adsorbents with high densities of metal chelating functionality, a feat that may help recover Pt from even the most dilute solutions and reduce Pt losses at various points throughout its life cycle.

Of a number of different classes of adsorbents to choose from, metal–organic frameworks (MOFs) are particularly attractive because they offer record-setting porosity combined with highly modular structures.^11^ Recent literature has identified several MOFs as promising adsorbents for the recovery of precious metals from various liquid streams.^12–14^ For instance, Zr-BDC-NH_2_ (alternatively known as UiO-66-NH_2_; BDC-NH_2_ = 2-aminobenzene-1,4-dicarboxylic acid)^15^ and Cr-BDC-NH_2_ (alternatively known as MIL-101(Cr)–NH_2_)^16^ were used for the recovery of Pt species. However, the Pt capacities were reportedly 100 mg g^−1^ and 150 mg g^−1^ at equilibrium concentrations of >1400 ppm for Cr-BDC-NH_2_ and Zr-BDC-NH_2_, respectively. To boost framework-metal affinity, Daliran *et al.* post-synthetically grafted metal chelating moieties to the linker in Zr-BDC-NH_2_, increasing the uptake of a targeted precious metal, Pd.^13^ Further, our group and others have recently demonstrated that polymer guests, grown inside MOF pores from monomer precursors, significantly enhance the uptake of various precious metals, like Au, Pd, and Ag.^17–21^ Similar to previous efforts aimed at post-synthetically modifying MOF linkers, we hypothesized that the polymers, which are trapped inside MOF pores, could also be post-synthetically modified with high densities of metal chelating functionality using simple organic chemistry techniques. This is particularly attractive because polymers are also known to enhance the chemical and mechanical stability of MOFs.^22,23^ To demonstrate this, we carried out post-synthetic modifications (PSM) on a known MOF-polymer composite, Fe-BTC/PDA.^24^ The selected MOF, Fe-BTC (also known as MIL-100), is robust, consists of Fe_3_(μ_3_-O) clusters interlinked *via* 1,3,5-benzenedicarboxylate ligands (BTC^3−^) and has a chemical formula of Fe_3_O(H_2_O)_2_OH(BTC)_2_.^25^ The MOF was infused with dopamine monomers that polymerize inside the pores *via* an anaerobic oxidation process that is triggered by redox-active Fe(iii) sites found inside the MOF pores. The host framework provides extrinsic porosity to PDA, making its metal-scavenging catechol and amine groups highly accessible by target metal ions that enter the MOF pores.^17,24^ Notably, we recently found that the PDA oligomers formed inside the MOF pores contain a higher density of primary amines when compared to bulk-PDA polymer formed using conventional methods. This implies that the MOF plays a strong role as a structure-directing agent during polymer formation.^26^ The higher density of primary amines in Fe-BTC/PDA can be favorable for the adsorption and reduction of Pt(iv),^15,27^ and the catechol units of PDA are known to both chelate and reduce metal ions.^28^ Additionally, PDA can be post-synthetically modified, with various functional groups, such as amines and thiols.^29^ For instance, small molecules can be grafted to the PDA backbone *via* Michael addition and/or Schiff base reactions.^23,30,31^ Given this, it was hypothesized that the PDA oligomer could also undergo PSM while inside the pores of Fe-BTC, possibly boosting the density and strength of adsorption sites present in the MOF-polymer composite and leading to improved capacities and removal efficiencies for targeted soft metal species, like Pt.

In the title work, thiol groups were post-synthetically grafted onto the backbone of PDA while inside of Fe-BTC, *via* a Michael addition reaction, thereby increasing the number of strong adsorption sites for Pt(iv) species in Fe-BTC/PDA. The chosen thiol was 2,3-dimercapto-1-propanol (DIP) due to available SH and OH groups that can readily react with PDA and chelate metal ions.^32^ Further, X-ray photoelectron spectroscopy (XPS) and *in situ* X-ray absorption spectroscopy (XAS) studies were used to elucidate the effect of the modified polymer on the adsorption/reduction process occurring during Pt(iv) extraction. By comparing the ratios of Pt(ii) and Pt(iv) on the surface and in the bulk of the MOF/polymer composite, it was found that Pt(iv) is more readily reduced in the pores of the composite, which is facilitated by the redox-active polymer inside the MOF pores. Finally, it was shown that the post-synthetic modification strategy can be used to graft various small molecular chelates beyond DIP to the polymer backbone, potentially paving the way to tune such MOF/polymer composite systems toward targeted metal separations in the future.

## Results and discussion

### Characterization of modified Fe-BTC composites

**Scheme 1:**
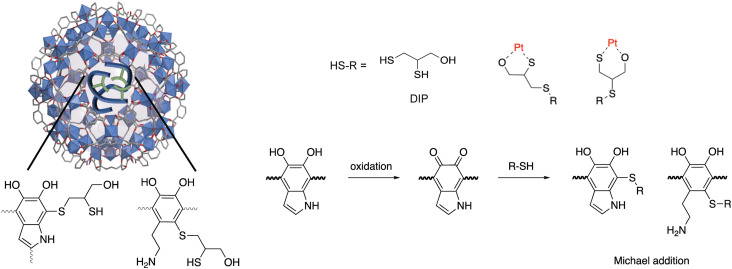
Schematic representation of two-step post-synthetic modification strategy involving *in situ* oxidative polymerization of dopamine in Fe-BTC followed by grafting thiols (DIP) as metal scavenging chelation groups to polydopamine.

**Fig. 1:**
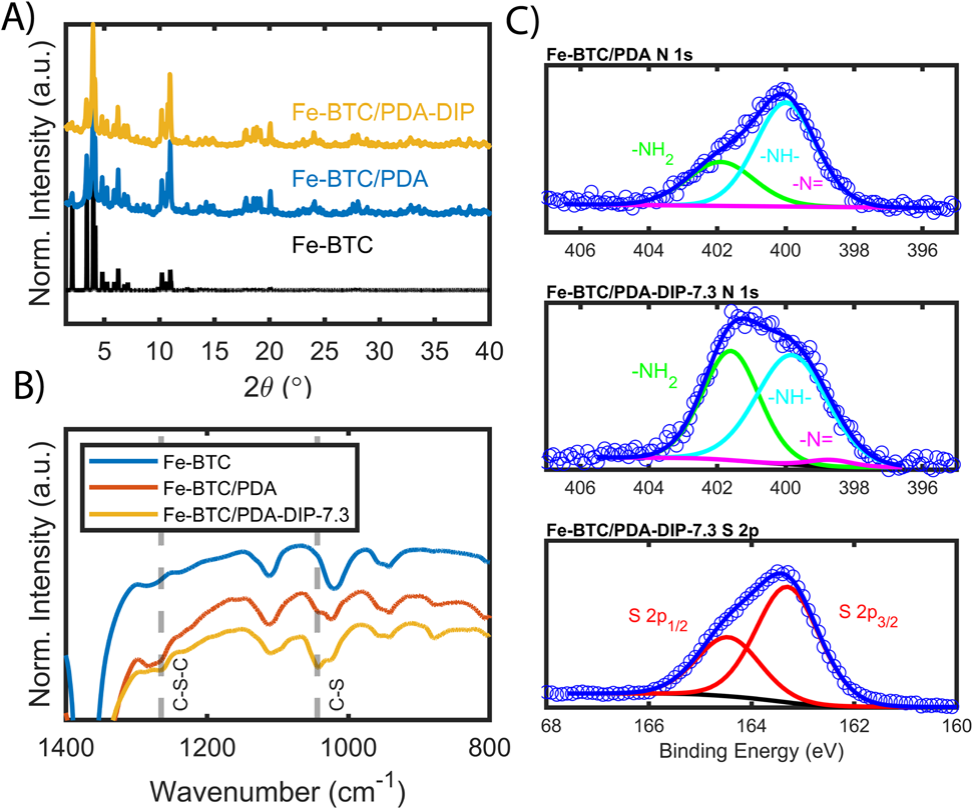
(A) shows the PXRD patterns of the simulated MOF structure, the as-synthesized Fe-BTC and the post-synthetically modified Fe-BTC/PDA-DIP-7.3. (B) FT-IR spectra of Fe-BTC, Fe-BTC/PDA, and Fe-BTC/PDA-DIP-7.3 with C–S–C and C–S vibration bands. (C) XPS spectra of the N 1s region of Fe-BTC/PDA and Fe-BTC/PDA-DIP-7.3 and the S 2p region of the latter.

Fe-BTC/PDA (12 wt% PDA) was synthesized *via* an *in situ* oxidative polymerization of dopamine inside the pores of Fe-BTC using a previously reported protocol.^24^ DIP was then grafted to the PDA using a Michael addition reaction (Scheme 1) (see Materials and methods in the ESI† for more information). The loading of DIP was varied to investigate the effect of the appended molecule on Pt(iv) adsorption. Powder X-ray diffraction (PXRD) patterns of the as-synthesized composites, referred to as Fe-BTC/PDA-DIP-*X* (where *X* = DIP wt% in the composite), confirmed the structural integrity of the MOF after all modifications (Fig. 1A and S1†). Additionally, the supernatant of the reaction mixture was analyzed with ICP-OES, and no Fe leaching was detected. On the contrary, a control experiment, where the bare Fe-BTC was soaked in a DIP solution in methanol, there was significant Fe leaching (Fig. S2†). This indicates that PDA improves the chemical stability of Fe-BTC; it is thought that the polymer could limit access to the Fe-based building units, inhibiting structural degradation of the MOF when in contact with DIP. Next, the relative ratios of Fe-BTC, PDA, and DIP were determined using elemental analysis (EA) and thermogravimetric analysis (TGA). Using EA, the weight percent of C, H, N, and S in the different composites were determined, allowing quantification of the PDA and grafted thiols separately. The results, summarized in Table S1†, show that the loading of DIP can be controlled by varying the relative ratios of the reagents in the reaction mixture. The calculated polymer loadings and the corresponding theoretical molecular formulas of the MOF/polymer composites also correlate well with TGA results (Fig. S3†).

Accordingly, N_2_ adsorption isotherms, shown in Fig. S4,† reveal that the surface areas of the composites were reduced sequentially as the quantity of grafted thiols increased (Brunauer–Emmet–Teller (BET) surface areas are listed in Table S1†). It should be noted that, for the two highest loadings of DIP, the thiols were added in excess relative to the monomeric units of PDA; despite this, the highest achieved DIP loading of 7.3 wt% corresponds to roughly a 1 : 0.85 ratio of monomeric dopamine to DIP units (see Table S1† for details). This suggests that the limiting factor for the DIP loading is the number of available grafting sites on the PDA backbone.

**Fig. 2:**
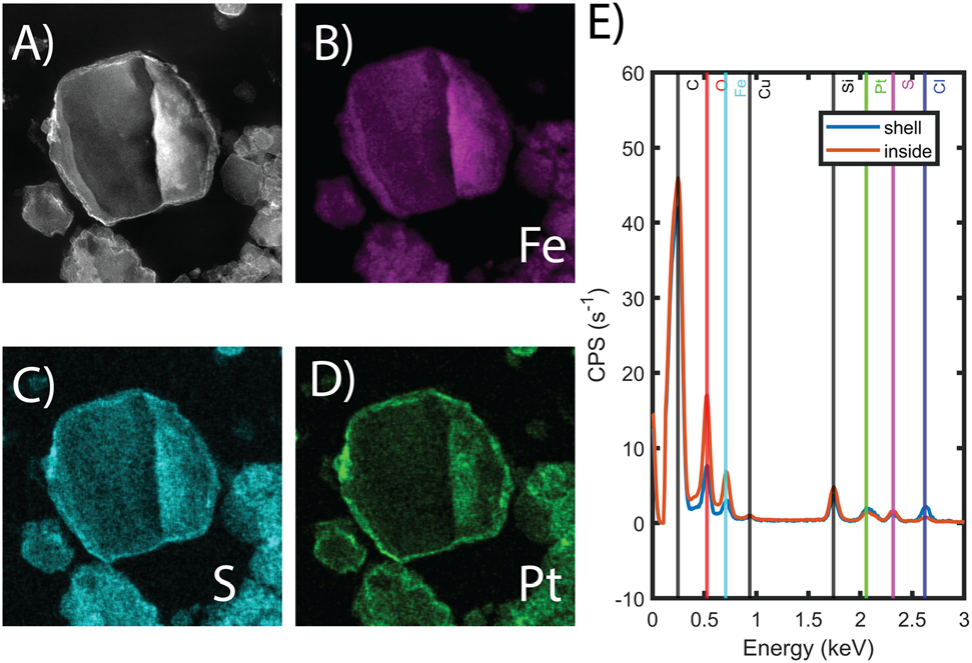
(A) HAADF-STEM images of sliced particle of Fe-BTC/PDA-DIP-7.3. (B–D) show the EDX elemental mapping for Fe, S and Pt, originating from the MOF/polymer. (E) shows the EDX spectra taken from the shell and the inside of the imaged particle.

FT-IR, mass spectrometry, XPS, and electron microscopy were used to assess the functionalization of the MOF. Fig. 1B shows the FT-IR spectra of the bare MOF, Fe-BTC/PDA, and Fe-BTC/PDA-DIP-7.3. Two peaks at 1044 and 1265 cm^−1^, which appear after the DIP modification, can be assigned to *ν*(C–S) and *ν*(C–S–C) vibrations, respectively.^33,34^ These peaks confirm the successful grating of DIP to PDA. Next, XPS of the N 1s region of Fe-BTC/PDA-DIP-7.3 was compared to Fe-BTC/PDA. The spectrum of Fe-BTC/PDA-DIP-7.3 has a higher relative intensity of primary amines (Fig. 1C), indicating that the functionalization results in an opening of the indoline/indole ring on the PDA backbone. This observation agrees with a previous report that indicates that the β carbon of the catechol unit in PDA is the most favorable site during a Michael addition.^35^ This naturally leads to the ring opening of the indoline/indole ring of PDA and, consequently, to a higher density of primary amines on the oligomeric structure. The S 2p region of the XPS spectra further confirms the successful grafting of DIP into the composite (Fig. 1C). Next, the polymeric units were assessed using electrospray ionization spectrometry (ESI-MS) before and after modification. For this, the polymeric guests, including both PDA and PDA–DIP were liberated from the host framework by dissolving the MOF in an EDTA solution, yielding the isolated polymers as a dispersion (see the ESI† for experimental details). Fig. S5† shows a digital photograph of PDA and PDA–DIP dispersed in the EDTA solution after MOF dissolution, and Fig. S6 and S7† show the obtained ESI-MS spectra of the isolated polymers labeled PDA and PDA–DIP. The highest molecular weight unit observed for the isolated PDA was 877 *m*/*z*, indicative of 6 monomeric units. Proposed structures that correspond to this mass are shown in Fig. S6.† After modification with DIP, higher molecular weight units are clearly observed (Fig. S7†). The highest mass was 1370 *m*/*z*, which could correspond to 6 monomeric units of dopamine (found in isolated PDA) modified with 4 units of DIP. Finally, scanning transmission electron microscopy (STEM) combined with energy dispersive X-ray (EDX) elemental mapping was used to probe the cross-section of the as-synthesized MOF/polymer composites. STEM images and elemental maps of Fe, S, and Pt (Fig. 2A–D) were obtained from Fe-BTC/PDA-DIP-7.3 particles embedded in an epoxy resin and serially sliced. Note that the sample was soaked in a 200 ppm Pt(iv) solution and the Pt mapping is discussed later. The data shows the successful addition of the DIP throughout the MOF (associated EDX spectra are shown in Fig. 2E and S8†). SEM images (Fig. S9†) show no sign of a separate bulk polymer phase within the MOF particles. Finally, RAMAN spectroscopy shows the absence of any Fe–S vibrational bands (expected to appear around 312 cm^−1^).^36^ On the contrary, the presence of Fe-catechol interactions are confirmed by three signature peaks at 550, 596, and 637 cm^−1^ (Fig. S10†).^37,38^ This data confirms that DIP is grafted to PDA rather than the Fe-based building unit of the MOF, while some of the dopamine units are bound to the Fe.

### Platinum adsorption experiments

**Fig. 3:**
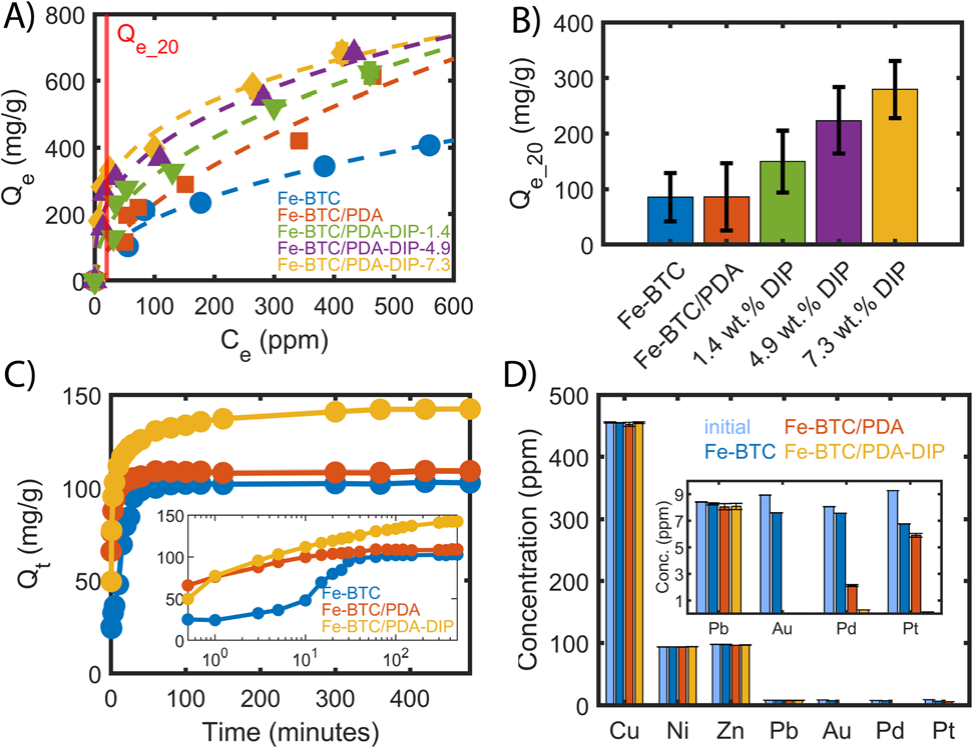
(A) Pt(iv) adsorption isotherm measured at 28 °C fitted to the Freundlich adsorption model comparing different DIP loadings. Each point was measured in triplicate and averaged. The dashed lines are the fitted Freundlich models, and the red line indicates the position at *C*_e_ = 20 ppm, adsorbent dosing = 0.5 mg ml^−1^, 24 hours). (B) *Q*_e_20 ppm_ value (uptake at an equilibrium concentration of 20 ppm) predicted form the modelled Freundlich adsorption isotherms. The values are calculated at an 80% confidence level. (C) Pt removal as a function of time (*C*_0_ = 75 ppm, adsorbent dosage = 0.5 mg ml^−1^), with the inset showing the evolution of *Q*_t_ on a logarithmic time-scale. (D) Pt(iv) adsorption of Fe-BTC/PDA-DIP-7.3 measured in a presence of competing ions. The inset shows the results zoomed in for the low-concentration ions. Adsorbent dosage = 1 mg ml^−1^, adsorption time = 4 hours. Note for (A, C, and D), some error bars are smaller than the diameter of the symbols.

Given successful grafting, the effect of the thiols on the Pt adsorption capacity and kinetics was investigated. Notably, the Pt(iv) uptake of the composites was investigated before and after DIP grafting. The aim was to assess how the grafted molecules contribute to the performance of Fe-BTC/PDA-DIP. For this purpose, the Pt uptake of Fe-BTC, Fe-BTC/PDA, and Fe-BTC/PDA-DIP was measured in solutions with varying Pt(iv) concentrations ranging from 100 to 700 ppm and an adsorbent loading of 0.5 mg ml^−1^ over 24 hours (Fig. 3A). Notably, Synchrotron PXRD patterns of Fe-BTC/PDA-DIP-7.3 indicate that the material maintains its crystallinity before and after Pt adsorption (Fig. S11†). The Pt uptake steadily increases with increasing DIP loading, showing that the grafted molecules indeed boost the density and/or strength of available adsorption sites. Considering that there are multiple adsorption sites, the Freundlich adsorption model (1), which is suitable for heterogeneous surfaces,^39^ was used to model the obtained data.

1 *Q*_e_ = *K*_F_ × *C*_e_^1/*n*^

The resulting fitting parameters can be found in the ESI (Table S2),† and the calculated *Q*_e_ for different equilibrium concentrations can be found in Tables S3–S7†. For comparison, the data of the bare Fe-BTC and Fe-BTC/PDA were added as well. Importantly, for the Fe-BTC/PDA-DIP series, the Pt adsorption isotherms become much steeper in the low concentration regime, below 100 ppm. This is important for recycling efforts as it can help minimize Pt loss from dilute waste streams, reduce the size of the adsorbent bed, and/or increase the length of time between services (*i.e.*, changing or regenerating the adsorbent). Accordingly, the Freundlich constant *K*_F_ and the parameter 1/*n* are steadily increasing with increasing amounts of grafted DIP (see Table S2†). *K*_F_ is related to the adsorption capacity and the parameter 1/*n* to the heterogeneity of the surface.^40,41^ The increase in the value of both parameters suggests that the adsorbent has a higher affinity to Pt and that the surface is becoming less homogenous in nature. The Pt uptake of all materials was calculated using the Freundlich fit at an equilibrium concentration of 20 ppm (*Q*_e_20_). The *Q*_e_20_ values, shown in Fig. 3B, are given with an 80% confidence level (see ESI,† Section 2.2). Notably, in this regime, Fe-BTC/PDA-DIP-7.3 offers a capacity that is ∼3 times higher than Fe-BTC or Fe-BTC/PDA. Moreover, Fe-BTC/PDA-DIP-7.3 also exhibits a record Pt capacity of 684 mg g^−1^ in the high concentration regime (Fig. 3A), which is the highest value reported to date for a MOF-based adsorbent.

To gain insight into the speed of the extraction process, the Pt removal efficiency was determined as a function of time. Fig. 3C shows the removal efficiency of Fe-BTC, Fe-BTC/PDA, and Fe-BTC/PDA-DIP-7.3. Importantly, Pt accumulates more quickly in Fe-BTC/PDA-DIP-7.3 than the others. Next, the kinetic adsorption data was fitted to a pseudo-second-order (PSO) adsorption model in Fig. S12† to determine the rate constants; the fitted parameters, shown in Table S8†, indicate that the polymer does not seem to slow down the adsorption rate. In fact, despite the pore filling with polymeric species, Fe-BTC/PDA-DIP has rate constant that is comparable to the bare framework (173.65 × 10^−5^ ± 2.5 × 10^−5^ and 200.79 × 10^−5^ ± 5.89 × 10^−5^ g mg^−1^ min^−1^, respectively) and a drastically improved equilibrium Pt uptake (143 mg g^−1^ and 104 mg g^−1^).

Finally, the Pt(iv) selectivity of Fe-BTC, Fe-BTC/PDA, and Fe-BTC/PDA-DIP-7.3 were assessed in a competitive environment that contained common competing ions (*i.e.*, Cu(ii), Ni(ii), Zn(ii), Pb(ii), Au(iii), and Pd(ii) at a pH = 3). These metals were chosen because they are prevalent in solutions used to leach metals from electronic waste and, hence, could potentially compete with Pt for adsorption sites under realistic conditions.^14,20,42,43^ As shown in Fig. 3D, Fe-BTC/PDA-DIP-7.3 effectively removed Pt and other precious metals (Au and Pd) without taking up any of the base metals, including Cu, Ni, or Zn. Surprisingly, only a small Pb uptake was observed, indicating a much higher affinity for the three precious metals. From the competitive adsorption data, distribution coefficients (Table 1) were calculated for Pb, Pt, Pd, and Au. The *K*_d_'s provide an indication each material's affinity towards a given metal and the ratio of two *K*_d_'s can be used to determine the selectivity factor *α*.^44^ Given minimal to no adsorption observed for Cu, Ni, and Zn, the *K*_d_'s for these metals were too small to calculate. Notably, the *K*_d_'s obtained from Fe-BTC indicate a relatively low affinity for all metals. However, upon modification with PDA, there is a stark increase in Fe-BTC's affinity towards precious metals with a selectivity trend that follows Au > Pd > Pt. In particular, the impressive affinity towards Au likely stems from the redox active polymer combined with the high reduction potential of AuCl_4_^−^ species. Next, grafting DIP to Fe-BTC/PDA drastically enhances the material's affinity towards Pt. This change can, in part, be explained by the hard soft acid base theory, where thiols are expected to favor the complexation of soft Lewis acids, like Pt(iv), over harder base metals.^45^ While the introduction of PDA and PDA-DIP improves the Pb uptake some, a much higher selectivity towards Pt is achieved with selectivity factors of *α* = 22, 13, and 1′539 for Fe-BTC, Fe-BTC/PDA, and Fe-BTC/PDA-DIP-7.3, respectively (*α* = *K*_d_^Pt4+^/*K*_d_^Pb2+^). Notably, it is not yet understood why the DIP offers such an improved affinity for Pt over that of Au and Pd; however, it is likely related to the stability of the resulting Pt–DIP complex.

**Table 1:** Distribution constants were calculated from the multielement adsorption experiment with initial concentrations of 10 ppm for precious metals and lead and excess of Cu (500 ppm), Ni, and Zn (100 ppm each). The errors are standard deviations from triplicated experiments

Binary mixture sample	*K*_d_ (Pb^2+^) [ml g^−1^]	*K*_d_ (Au^3+^) [ml g^−1^]	*K*_d_ (Pd^2+^) [ml g^−1^]	*K*_d_ (Pt^4+^) [ml g^−1^]
Fe-BTC/PDA-DIP-7.3	41 ± 31	142′069 ± 8′093	28′470 ± 238	62′845 ± 0
Fe-BTC/PDA	45 ± 31	209′788 ± 17′535	3′051 ± 162	331 ± 36
Fe-BTC	16 ± 11	174 ± 1	134 ± 0	273 ± 29

Given the high performance of Fe-BTC/PDA-DIP-7.3 towards Pt extraction, the material was compared to several other reported MOFs and a commercial resin known as Amberjet 4200 (see Table S9†). From the materials found in the literature, Fe-BTC/PDA-DIP-7.3 is superior to all in terms of capacity and *K*_d_. For instance, when tested under similar conditions (*C*_0_ = 70–100 ppm Pt^4+^), Fe-BTC/PDA-DIP-7.3 has a ∼40% higher adsorption capacity and a distribution coefficient that is ∼8 times higher than Amberjet 4200; we believe this stems from the strong interactions between the DIP and Pt.

Finally, the cyclability of Fe-BTC/PDA-DIP-7.3 was tested after Pt adsorption. Notably, the material's very high affinity for the metal makes it difficult to find adequate regeneration procedures that do not concomitantly destroy the MOF, which is acid-sensitive (pH < 3). Nonetheless, preliminary tests were done to assess Pt desorption from the composite using a regeneration solution composed of 0.8 M thiourea in 0.5 M HCl. Fig. S13A† shows the results from three adsorption–desorption cycles. While full Pt desorption was achieved in the first cycle, a significant amount of Fe leaching was also detected, indicating limited stability of the framework in the acidic regeneration conditions. The PXRD pattern also shows that the composite becomes rather amorphous after repeated adsorption–desorption cycles (Fig. S13B†), and there is an ∼20% drop in the quantity of Pt extracted in the second cycle. This indicates that the regeneration process should be optimized. While we will make an effort to search for milder regeneration conditions in the future, it is noted that the PSM reported in this work could also be readily extended to other more stable porous supports that allow one to employ harsher regeneration conditions. Finding a balance between strength of adsorption and ease of regeneration are important factors to consider as we move towards more sustainable separation processes in the future.

### Adsorption mechanism

To show that the Pt is distributed throughout the MOF-polymer composite, FE-BTC/DIP 7.3 was soaked in a 200 ppm platinum solution, then embedded in an epoxy resin, serially sliced using a microtome (≈80 nm), and imaged. Fig. 2D shows the STEM EDX elemental map obtained from the sample. From the image, it is clear that the platinum can diffuse throughout the MOF/polymer composite. Notably, there appears to be a higher density of Pt on the surface than in the bulk. While we cannot rule out diffusion limitations, it should be noted that any polymer that forms on the external surface will naturally be denser than the polymer formed in the pores as also indicated by the S map in Fig. 2C. Thus, it is not surprising that the Pt signal close to the surface is more intense than that found inside.

**Fig. 4:**
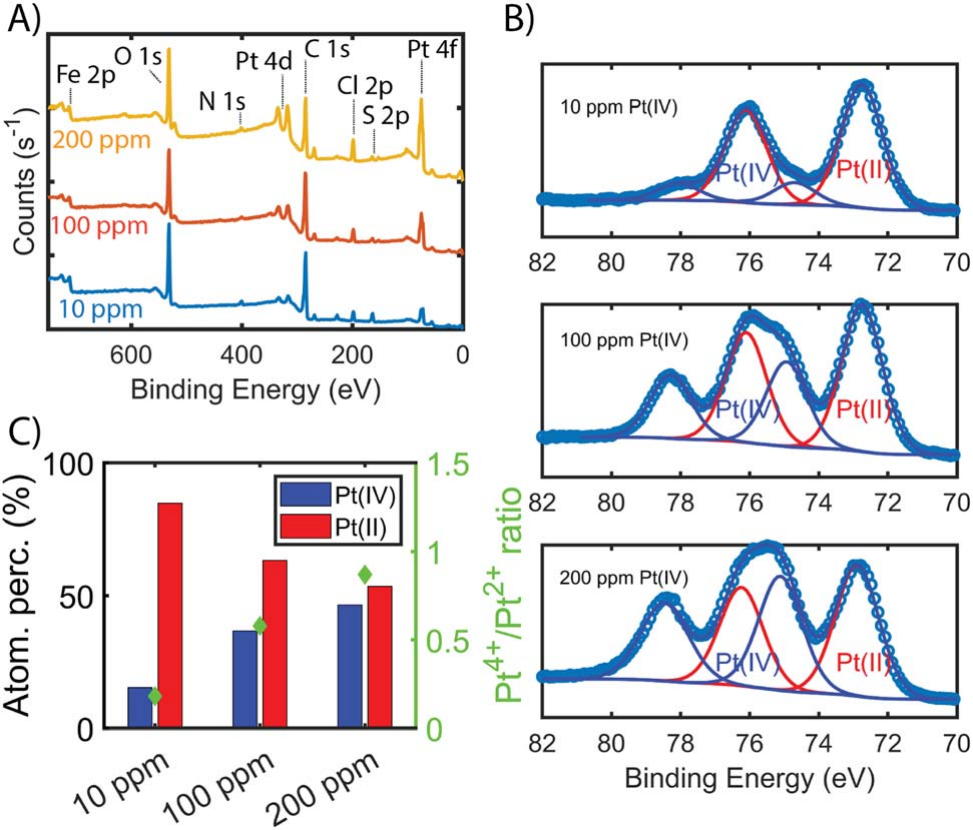
(A) shows the wide XPS scan with elemental labelling of Fe-BTC/PDA-DIP-7.3 soaked in Pt(iv) solutions with increasing concentration (10, 100, 200 ppm, 1 mg ml^−1^, 6 hours adsorption time). (B) Fitted Pt 4f XPS data for Fe-BTC/PDA-DIP-7.3 soaked in Pt(iv) solutions with increasing Pt concentration. (C) The relative percentage of adsorbed Pt(iv) and Pt(ii) are calculated from the synthetic peak areas of the fitted Pt(iv) and Pt(ii) 4f_7/2_ and 4f_5/2_ doublets in (A).

**Fig. 5:**
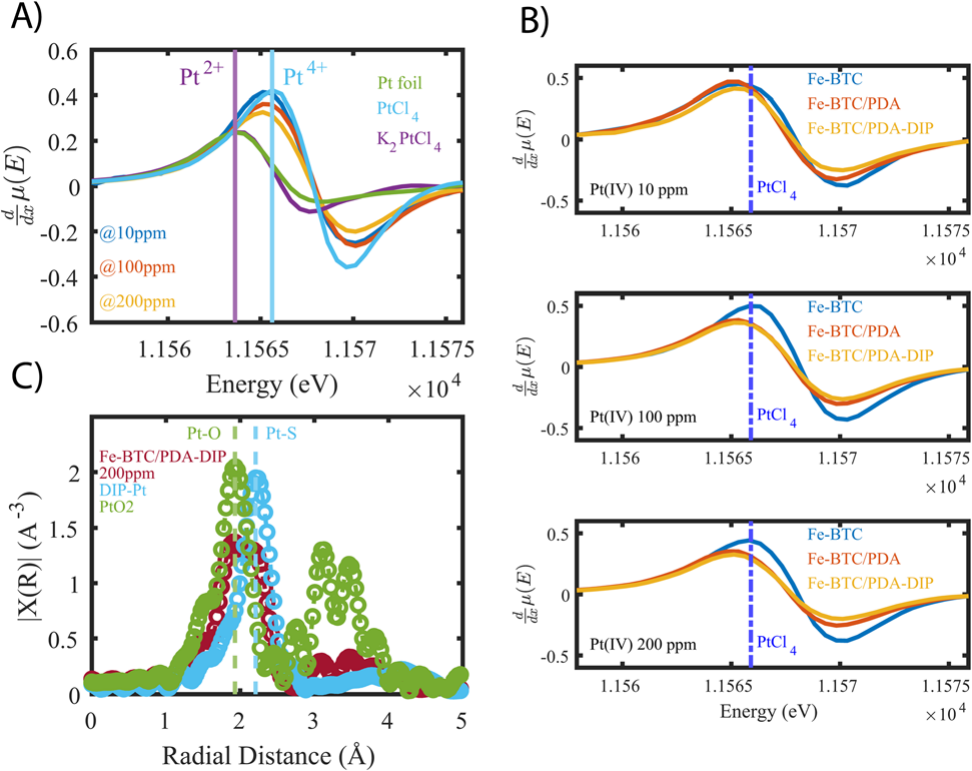
(A) derivatives of X-ray absorption near edge structures (XANES) of Fe-BTC/PDA-DIP-7.3 loaded with increasing concentrations of platinum, compared to standards with varying oxidations states (0, ii, and iv). (B) Compares XANES derivatives of the bare MOF to those of Fe-BTC/PDA and Fe-BTC/PDA-DIP at different Pt saturation points. For reference, the peak position of the PtCl_4_ edge is marked as the dotted blue line. (C) Fourier Transformed (FT) of the *k*^2^ weighted EXAFS data (corrected for phase-shift. FT range: *k* = 3–14 Å^−1^, see Fig. S27A†) of Fe-BTC/PDA-DIP-7.3 soaked in a 200 ppm Pt solution, compared to PtO_2_ and a DIP–Pt complex.

Next, we aimed to gain insight into the extraction mechanism; to do so, synchrotron XAS and laboratory-based XPS were used. Briefly, Fe-BTC/PDA-DIP-7.3 was soaked in solutions with varying Pt(iv) concentrations (10, 100, and 200 ppm) and then subjected to XPS and XAS analysis to determine changes in the oxidation state of Pt(iv) upon adsorption at the different saturation levels. Firstly, the wide scan XPS data in Fig. 4A clearly shows an increasing count rate for Pt (4f and 4d) due to increased Pt adsorption. In the fitted Pt 4f spectra, Fig. 4B, two doublets representing Pt(ii) and Pt(iv) can be seen (see Table S10† for the exact binding energy, BE). From this data, the Pt(iv) : Pt(ii) ratios were calculated and determined to be 15 : 85, 37 : 63, and 47 : 53 (Fig. 4C). The presence of Pt(ii) is not surprising, as we previously observed that redox-active polymers grown inside MOFs could readily reduce metals,^17,19,24^ significantly enhancing extraction performance. It appears that with exposure of Fe-BTC/PDA-DIP-7.3 to higher concentrations of Pt, the relative contribution of Pt(iv) increases relative to Pt(ii). Notably, at the lowest concentration, 10 ppm, most of the Pt(iv) is reduced to Pt(ii), whereas at higher saturation levels, the amount of unreduced Pt(iv) steadily rises. Given that XPS is a surface-sensitive technique, synchrotron XAS studies were also conducted to provide additional insight into the Pt oxidation states throughout the entire material. The Pt 4f photoelectrons measured in XPS originate within the first tens of angstroms of the particle surface,^46^ whereas in standard transmission XAS, the intensity of X-rays before and after the substrate is used to generate spectra. Thus, XAS is representative of the bulk material in the beam.^47^ If the polymer, which is predominately responsible for the Pt reduction, is mainly located inside the crystalline MOF particles, it was expected that there might be differences in the calculated ratios from the two different characterization techniques. For the Pt L-III edge, XANES of different oxidation states overlap; while this makes determining the Pt(iv) : Pt(ii) ratio difficult, lower oxidation states of Pt will shift the edge to lower energies and cause a reduction in the white line intensity (decrease in the peak maximum of the edge). Fig. 5A shows the derivative of the Pt L-iii edge in Fe-BTC/PDA-DIP-7.3 with different Pt loadings compared with Pt standards having oxidation states of iv, II, and 0. For Fe-BTC/PDA-DIP-7.3, the peak maximum is shifted to lower energies, and the intensity decreases upon increasing the Pt loading; this indicates that the relative amount of Pt(ii) compared to Pt(iv) increases with increasing Pt loading. Interestingly, the opposite trend is observed in the XPS data (Fig. 4C), likely indicating that the species responsible for Pt reduction close to the surface are more quickly saturated than those in the bulk.

Fig. 5B also compares the XANES data collected from Fe-BTC, Fe-BTC/PDA, and Fe-BTC/PDA-DIP-7.3 after exposure to 10, 100, and 200 ppm Pt(iv) solutions. The position of the PtCl_4_ (100% Pt(iv)) edge maximum is highlighted in all three experiments as a dashed blue line. It appears that the edge position for Fe-BTC shifts to higher energies, indicating that with higher Pt loadings, the amount of Pt(iv) relative to Pt(ii) increases. On the other hand, for the MOF/polymer composites, we see a shift to lower energies, indicating that the amount of Pt(ii) relative to Pt(iv) increases (as already shown in Fig. 5A). This shows that the presence of the polymers clearly leads to more Pt reduction. The Pt reduction observed in Fe-BTC could be the consequence of Fe(ii) sites present in the MOF structure,^48–50^ which have been previously shown to reduce Pt(iv) in solution while irradiated with visible light.^51^ We do observe the presence of Fe(ii) by high-resolution XPS data collected on Fe-BTC/PDA-DIP-7.3, and the Fe 2p region reveals that the Fe(ii) peaks decreased in intensity after the material is exposed to a 10 ppm solution of Pt(iv) (see Fig. S14 and Table S11†). Since the density of these Fe(ii) sites is expected to be generally low in Fe-BTC, it is also expected that the amount of Pt(iv) that the bare MOF can reduce is limited. The XANES data clearly shows that the presence of PDA and PDA-DIP leads to more Pt(iv) reduction. Further, Fe-BTC/PDA-DIP-7.3 consistently shows lower white line intensity than Fe-BTC/PDA, suggesting that the thiol-modified polymer improves Pt(iv) reduction compared to non-modified PDA.

Next, XAS and XPS were used to probe the coordination environment of the adsorbed Pt. As a control, free DIP was first added to a Pt(iv) solution, allowing the formation of DIP–Pt complexes (see ESI†). XPS of the obtained complex revealed that it consists mainly of Pt(ii) species (see Fig. S15†), showing that the thiols readily reduce Pt(iv) to Pt(ii). Further, extended X-ray absorption fine structures (EXAFS) (Fig. 5C) of the precipitate reveal an atomic pair with a radial distance matching that of Pt–S bonds.^52,53^ Pt–Cl, which has a similar bond distance,^54^ can be excluded as a candidate since XPS showed no Cl signal (Fig. S15†). Next, EXAFS data was collected for Fe-BTC/PDA-DIP-7.3. The data reveals two distinct peaks at 1.93 Å and 2.23 Å, which can be attributed to a Pt–O (or, indistinguishable, to Pt–N) and a Pt–S bond (Fig. 5C); this indicates that the DIP is likely reducing Pt(iv) and chelating Pt(ii) and explains why more Pt(iv) is reduced in Fe-BTC/PDA-DIP-7.3 relative to Fe-BTC/PDA. Notably, the EXAFS data of Fe-BTC/PDA-DIP-7.3 also indicates that the main peak in the coordination shell of adsorbed Pt is coming from Pt–O/N bonds, which likely stems from coordination to catechols, quinones, and amines on the PDA backbone.^54^ Wavelet transform (WT)-EXAFS in Fig. S16† provides more support for the existence of two separate and distinguishable Pt–O and Pt–S bonds. As further support, FT-IR data was collected for Fe-BTC/DIP-7.3 before and after Pt loading. The data reveals that the vibrational bands corresponding to C=O stretching increase after Pt adsorption (1710 cm^−1^), while bands coming from O–H stretching modes (573 cm^−1^) decrease (Fig. S17†). This can be explained by catechol groups becoming oxidized thereby transforming into quinone groups. Thus, this data supports the idea that PDA is involved in reducing Pt(iv) to Pt(ii) through its hydroxyl groups. Notably, FT-IR data also reveals changes in bands corresponding to C–N bonds observed at 877 cm^−1^, indicating that the amine groups in PDA also interact with Pt. XPS further supports this, revealing a shift in the N 1s region upon Pt adsorption (Fig. S18†).

**Fig. 6:**
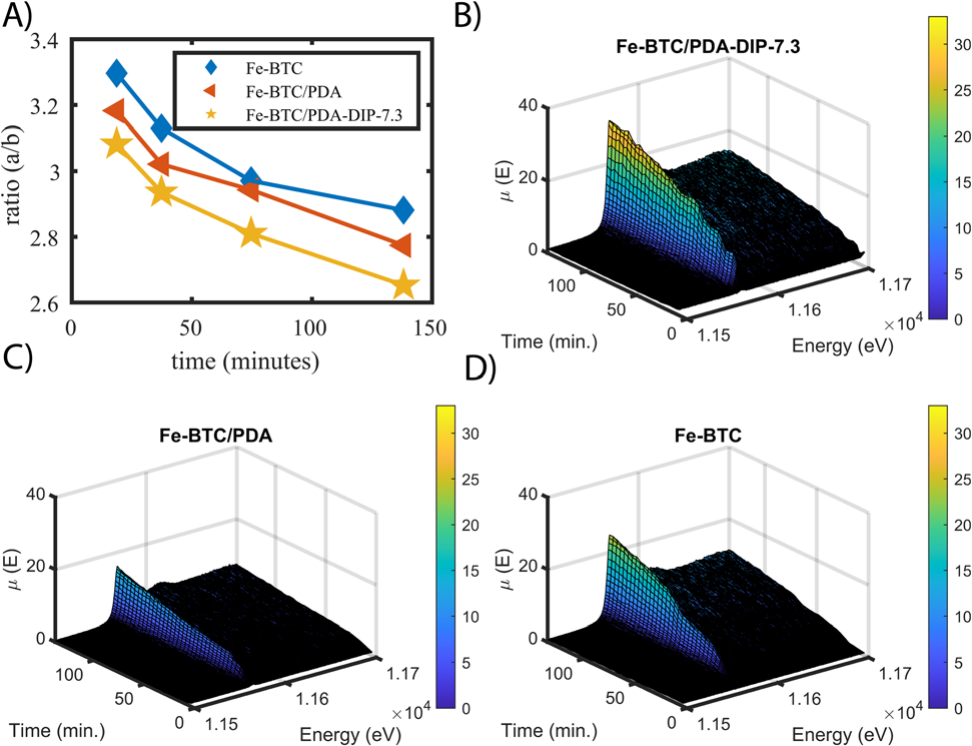
(A) shows the calculated ratio *a*/*b* Fe-BTC, Fe-BTC/PDA and Fe-BTC/PDA-DIP-7.3 at different time points of the *in situ* XANES measurements. (B) Fluorescence X-ray absorption spectra of the platinum edge measured *in situ* under a continuous flow of 100 ppm Pt(iv) solution through a fixed adsorbent bed containing Fe-BTC/PDA-DIP-7.3 and (C) Fe-BTC/PDA, and (D) Fe-BTC.

It has been hypothesized that redox reactions play a key role in boosting the precious metal extraction efficiency of several MOF/polymer composites and other adsorbents.^55^ However, all data presented to date was collected *ex situ*, making it impossible to see if such reduction reactions are occurring on a time scale that is relevant to the extraction process. Given this, we set out to assess the Pt oxidation state *in situ* for the first time during continuous flow experiments, which are closer to what might occur in real-world applications.^56^ For this purpose, XAS data were collected as a function of time, using a fixed bed column set-up that was placed in the beam path under a continuous flow of 100 ppm Pt(iv) solution. From this data, the relative differences in the Pt(iv) : Pt(ii) ratios and total Pt accumulation can be observed, allowing us to compare the three materials' performance under flow. In this experiment, XANES data was simultaneously collected in both transmission and fluorescence mode for Fe-BTC, Fe-BTC/PDA, and Fe-BTC/PDA-DIP-7.3 (fluorescence XAS generally gives better signals for dilute samples; see ESI† for more details). By comparing ratios of the white line intensities (*a*) over the post-edge minima (*b*) obtained from the transmission XANES data (see Fig. S19† for a detailed explanation),^57^ one can gain insight into the relative differences in the Pt(iv) : Pt(ii) ratios. Using this approach, the *a*/*b* ratio was determined at different time points. High values of (*a*/*b*) indicate higher Pt(iv) : Pt(ii) ratios. The selected normalized XANES are shown in Fig. S20.†Fig. 6A shows that the Pt(iv) : Pt(ii) ratio is lower for both MOF/polymer composites, indicating that the amount of reduced Pt increases during Pt accumulation inside the pores relative to Fe-BTC. This observation agrees with the trend observed in the *ex situ* experiments (Fig. 5B). Additionally, it shows that the reduction indeed occurs on a timescale similar to that of the adsorption process.

In Fig. 6B–D, the non-normalized fluorescence XANES data obtained from Fe-BTC/PDA-DIP-7.3, Fe-BTC/PDA, and Fe-BTC are plotted as a function of scan number. The intensity of the absorption step is proportional to the amount of absorbed species (Pt in this case). However, the Pt oxidation state can influence the white line intensity, so comparisons between the three samples must be made with caution. Despite this, after plotting the white line peak intensity as a function of scan number (Fig. S21†), we can still conclude that the total amount of Pt in Fe-BTC/PDA-DIP-7.3 is higher throughout the flow through the experiment. Hence, Pt must be accumulating faster in this material than the others. This conclusion can be drawn because it is also known that the Pt(iv) : Pt(ii) ratio is lowest for this material relative to the others. On the other hand, the lower white line intensity of Fe-BTC/PDA when compared to Fe-BTC is likely due to the presence of more Pt(ii) in Fe-BTC/PDA rather than less Pt accumulating in the sample (since batch kinetic experiments revealed a faster Pt(iv) uptake with a slightly higher *Q*_e_ in Fe-BTC/PDA compared to Fe-BTC, Fig. 3C).

In summary, the *in situ* XAS data shows that the total amount of accumulated Pt and the quantity of reduced Pt are greatest in the case of Fe-BTC/PDA-DIP-7.3. This demonstrates that decorating the polymer with chelating thiols drastically improves the material's Pt extraction efficiency under continuous flow. The data also shows that the reduction process is occurring on a time scale similar to the adsorption process and hence supports the idea that reduction plays a key role in metal recovery.

### Extension of post-synthetic modification strategy

Altering the structure of the grafted thiols could be an easy way to develop novel composites with even better Pt recovery properties in the future. Given this, the PSM process developed here was extended to other chelating molecules beyond DIP. For this, a set of thiol-containing molecules, including 2,2-thiodiethanethiol (TDIET), cysteine (CYS), and 2-aminoethanethiol (AET) (structures are shown in Scheme S1†) were selected, and the PSM was again carried out inside of Fe-BTC/PDA. Elemental analysis confirms the successful grafting of these molecules as the amount of sulfur in each sample was substantially increased (Table S12†). The different composites were also tested for Pt removal from a 90 ppm Pt(iv) solution. The results, shown in Fig. S22,† indicate that all three composites offer a significantly higher Pt uptake than Fe-BTC/PDA. In fact, there is a 247, 216, and 208% improvement in capacity for TDIET, CYS, and AET, respectively. Considering that Fe-BTC/PDA-TDIET has the best performance of the three, a more extensive characterization of this particular material was performed. Firstly, no Fe leaching was observed during the grafting, and the MOF crystal structure remained intact after the modification (Fig. S23 and S24†). Further, HAADF-STEM images and elemental mapping showed that TDIET is distributed throughout the composite particles (Fig. S25†). Notably, these three thiol-based chelates, including TDIET, CYS, and AET, were selected for this study because sulfur is not found in the MOF, providing an easy way to probe the success of the PSM. However, this chemistry can be readily extended to other chelates having different Lewis base functionality, like amines, allowing further enhancement or tuning of the composite performance toward other valuable and critical metals.

## Conclusion

In this work, we demonstrated that various thiols could be readily grafted to the backbone of polydopamine (PDA) *via* Michael addition reactions, which were carried out inside of a porous metal–organic framework, known as Fe-BTC (also MIL-100). This novel strategy produces high densities of strong adsorption sites inside of Fe-BTC/PDA, which boosts the composite's affinity towards Pt(iv) species. In short, by grafting 2,3-dimercapto-1-propanol (DIP) to Fe-BTC/PDA, the adsorption capacity for Pt(iv) was successfully increased to a record value of 640 mg per gram. Even more importantly, the composite, referred to as Fe-BTC/PDA-DIP, has an impressive Pt(iv) uptake in the low concentration regime <20 ppm, which is highly relevant to Pt recovery from waste streams. In fact, the capacity at an equilibrium concentration of 20 ppm (280 mg g^−1^) was improved by over a factor of three when compared to Fe-BTC/PDA and Fe-BTC. Further, Fe-BTC/PDA-DIP displays an exceptional selectivity towards precious metals over base metals, including Pb, Zn, Ni, and Cu. The material also offers a higher Pt capacity than a commercial resin, Amberjet 4200, which is commonly employed in Pt recovery. Last, using advanced spectroscopic techniques, including *in situ* XAS, we have provided evidence that the DIP-modification altered the redox activity of the polymer as it leads to larger quantities of reduced Pt(ii) species inside the MOF pores when compared to Fe-BTC and Fe-BTC/PDA. While we previously demonstrated that redox-active polymers, grown inside MOF pores, could significantly improve the MOF capacity during precious metal recovery, here we show for the first time, *via in situ* XANES, that the reduction of the precious metal is occurring on a time scale that is relevant to the recovery process carried out under continuous flow.

In the future, it is hoped that this work will motivate the design of many new MOF/polymer composites. For instance, by altering the composite building blocks (including the MOF, polymer, or chelating molecules), one can easily envision a host of new porous materials whose function is fine-tuned towards targeted metals. Additionally, we emphasize the benefit of using an array of different characterization techniques to better understand the structure-derived function of such materials. Finally, while having a high affinity is pertinent to boost adsorbent capacity and selectivity towards a targeted metal, particularly when that metal is found in low concentrations and highly competitive environments, we must also better understand the intricate interplay between strength of binding, regeneration conditions, and overall adsorbent stability as these factors will play a key role in the overall cost of the separation processes. Such knowledge is key for designing superior adsorbents that will make critical metal recovery from waste streams more feasible and, hence, help create a more circular metal economy, which is needed to forge the future energy transition.

## Data availability

Data for this paper, including raw files for all the plots in the manuscript and the ESI,† are available on Zenodo at https://doi.org/10.5281/zenodo.10056556

## Author contributions

T. Schertenleib and W. L. Queen designed the work, T. Schertenleib synthesized the materials and performed adsorption experiments and most material characterization and data analysis. V. V. Karve and M. Asgari did material characterization and helped design synchrotron experiments and collect XAS data. D. Stoian did XANES and EXAFS analysis. M. Mensi helped with XPS measurements and analysis. E. Oveisi performed HAADF-STEM characterization. T. Schertenleib, V. V. Karve, M. Asgari, O. Trukhina, and W. L. Queen wrote the manuscript.

## Conflicts of interest

There are no conflicts to declare.

## Supplementary Material

SC-015-D4SC00174E-s001
