# Supplementary material for: A post-synthetic modification strategy for enhancing Pt adsorption efficiency in MOF/polymer composites
Source: Chem Sci. 2024 May 10;15(22):8323–33. doi: 10.1039/d4sc00174e (PMC11151820; doi:10.1039/d4sc00174e)
Supplement: SC-015-D4SC00174E-s001 [file SC-015-D4SC00174E-s001.pdf]

Electronic Supplementary Material (ESI) for Chemical Science.
This journal is © The Royal Society of Chemistry 2024

Supporting Information

A Post-Synthetic Modification Strategy for Enhancing Pt Adsorption Efficiency in MOF/Polymer Composites

*Till Schertenleib, Vikram V. Karve, Dragos Stoian, Mehrdad Asgari, Olga Trukhina, Emad Oveisi, Mounir Mensi, Wendy L. Queen**

T. Schertenleib, V.V. Karve, M. Asgari, O. Trukhina, M. Mensi, W.L. Queen
Institue of Chemical Sciences and Engineering (ISIC)
École Polytechnique Fédérale de Lausanne (EPFL)
CH-1951 Sion, Switzerland
E-mail: wendy.queen@epfl.ch

D. Stoian
Swiss-Norwegian Beamlines
European Synchrotron Research Facilities (ESRF)
BP 220, Grenoble, France

M. Asgari
Department of Chemical Engineering and Biotechnology
University of Cambridge
CB3 0AS Cambridge, UK

E. Oveisi
Interdisciplinary Center for Electron Microscopy
École Polytechnique Fédérale de Lausanne (EPFL)
CH-1015 Lausanne, Switzerland

Contents

1 Materials and Methods **2**

1.1 Synthesis protocols 2

1.2 Adsorption experiments 5

1.3 Characterization 6

1.4 XANES and EXAFS measurements 7

2 Supplementary Figures and Tables **8**

2.1 Material characterization 8

2.2 Adsorption mechanism 14

2.3 Extension of the post-synthetic modification strategy 25

1 Materials and Methods

All chemicals used in this study were commercially purchased and used without any further purification. Iron(III) chloride hexahydrate (97.0-102.0 %, 5 Kg) was purchased from Alfa Aeser, Fisher Scientific AG. 1,3,5-Benzenetricarboxylic acid (98 %, 500 g) was purchased from ABCR GmbH. 2,3-Dimercapto-1-propanol (98+ %, 10 ml), 2,2'-thiodiethanethiol (90 %, 10 ml), zinc(II) chloride (98+%, 100 g), nickel(II) chloride hexahydrate (Biograde, 100 g) were purchased from Sigma-Aldrich. L-cysteine (98+ %, 5 g) was purchased from Fischer Scientific AG. 2-aminoethanethiol (99 %, 1 g), dopamine hycrochloride (98 %, 100 g), Ethylenediaminetetraacetic acid disodium salt (95 %, 500 g) were purchased from Chemie Brunschwig AG. cCpper(II) chloride hexahydrate (99 %, 250 g) was purchased from Acros Organics. Platinum(IV) chloride (PremiumARTM, crystalline soluble, 99.99 %, 1 g), potassium tetrachloroplatinate(II) (99.9 %, 1 g) were purchased from abcr GmbH. Sodium hydride (dry, 95 %, 10 g) was purchased from IVALUA.

1.1 Synthesis protocols

1.1.1 Synthesis of Fe-BTC

9.72 g of iron(III) chloride hexahydrate, 3.36 g of trimesic acid and 120 mL of distilled water were loaded in a 180 ml Teflon autoclave. The reaction mixture was heated to 130 °C for 72 hours. After the reaction was cooled down to room temperature the orange solid was filtered under vacuum and washed with copious amounts of water and methanol. The resulting powder was loaded into a double thickness Whatman cellulose extraction thimble and underwent Soxhlet purification with methanol for 24 hours. The sample was subsequently dried under vacuum overnight to yield 4 g of a light orange powder.

1.1.2 Synthesis of Fe-BTC/PDA

Fe-BTC/PDA was synthesized via in-situ oxidative polymerization of free base dopamine in Fe-BTC. Typically, 2 g of Fe-BTC were activated in a 500 ml two-neck round bottom flask under vacuum at 150 °C for 12 hours and then cooled down to room temperature. Separately, 800 mg of free base dopamine was added to a 500 ml two-neck round bottom flask and transferred to a glove box. Under a protective nitrogen atmosphere, the free base dopamine was dissolved in 400 ml of anhydrous methanol and transferred under nitrogen to the activated Fe-BTC. The dopamine solution was added to the activated MOF via a steel cannula. For this, the flask containing the Fe-BTC was briefly flushed with N2, while the flask containing the dopamine solution was pressurized with N2. By slowly pulling vacuum in the Fe-BTC flask, the dopamine solution flows to the Fe-BTC, and a rapid color

change to a very dark blue is observed. The reaction is kept stirring under nitrogen for 24 hours, followed by centrifugation and subsequently washing with methanol, and finally vacuum dried for 6 hours. The as-prepared Fe-BTC/PDA served as a starting material for the following post-synthetic modification (PSM) with thiols. For comparison with the later post-synthetically modified samples, a part was separated and underwent Soxhlet extraction with methanol for 24 hours followed by vacuum drying at room temperature (RT).

1.1.3 Post-synthetic modification of Fe-BTC/PDA with DIP

For the PSM, 300 mg of Fe-BTC was dispersed in 60 ml of methanol in a 100 ml one-neck round bottom flask. To this, 90 µl of triethylamine was added and the mixture was sonicated for 10 minutes followed by stirring open to the air for 50 minutes. Then, different equivalents of 2,3-dimercapto-1-propanol (DIP) (129, 290, 579, and 965 µmol) relative to the PDA amount were added. The reaction was kept stirring under air at room temperature for 12 hours. Then, the solid powder was separated with centrifugation, washed three times with methanol, and vacuum-dried for 3 hours. The product underwent Soxhlet extraction with methanol for 24 hours and was finally vacuum-dried at RT. The relative ratios of PDA and DIP, respectively, were determined with elemental analysis (N wt.% allows quantification of PDA and S wt.% allows quantification of DIP). According to their DIP and TDIET wt.%, the resulting composites were labeled Fe-BTC/PDA-DIP-1.4, Fe-BTC/PDA-DIP-2.4, Fe-BTC/PDA-DIP-4.9, and Fe-BTC/PDA-DIP-7.3.

1.1.4 Post-synthetic modification of Fe-BTC/PDA with TDIET

For the PSM, 150 mg of Fe-BTC was dispersed in 30 ml of methanol in a 50 ml one-neck round bottom flask. To this, 45 µl of triethylamine was added and the mixture was sonicated for 10 minutes followed by stirring open to the air for 50 minutes. Then, 482.5 µmol of 2,2'-thiodiethanethiol were added. The reaction was kept stirring under air at room temperature for 12 hours. Then, the solid powder was separated with centrifugation, washed three times with methanol, and vacuum-dried for 3 hours. The product underwent Soxhlet extraction with methanol for 24 hours and was finally vacuum-dried at RT.

1.1.5 Post-synthetic modification of Fe-BTC/PDA with cysteine

Due to the low solubility of cysteine in methanol, the reaction procedure was slightly changed. Cysteine (58.5 mg, 482.5 µmol) was added to a 100 ml round bottom flask and dissolved in 60 ml of a water:ethanol (50:50, vol.) mixture and heated to 50 °C. Then, 150 mg of Fe-BTC/PDA was added and the reaction was kept stirring at 50 °C for 12 hours. To avoid any solvent evaporation, a reflux condenser was connected to the flask. Then, the solid

powder was separated with centrifugation, washed three times with ethanol, and vacuum dried for 3 hours. The product underwent Soxhlet extraction with ethanol for 24 hours and was finally vacuum-dried at RT.

1.1.6 Post-synthetic modification of Fe-BTC/PDA with 2-aminoethanethiol

For the PSM, 150 mg of Fe-BTC was dispersed in 30 ml of methanol in a 50 ml one-neck round bottom flask. To this, 45 µl of triethylamine was added and the mixture was sonicated for 10 minutes followed by stirring open to the air for 50 minutes. Separately, 482.5 µmol (37.2 mg) of 2-aminoethanethiol were dissolved in 5 ml of methanol and then added to the reaction mixture. The reaction was kept stirring under air at room temperature for 12 hours. Then, the solid powder was separated with centrifugation, washed three times with methanol, and vacuum dried for 3 hours. The product underwent Soxhlet extraction with methanol for 24 hours and was finally vacuum-dried at RT.

1.1.7 Post-synthetic modification of Fe-BTC/PDA with 1,2-diethanethiol

For the PSM, 150 mg of Fe-BTC was dispersed in 30 ml of methanol in a 50 ml one-neck round bottom flask. To this, 45 µl of triethylamine was added and the mixture was sonicated for 10 minutes followed by stirring open to the air for 50 minutes. Then, 482.5 µmol of 1,2-diethanethiol were added. The reaction was kept stirring under air at room temperature for 12 hours. Then, the solid powder was separated with centrifugation, washed three times with methanol, and vacuum-dried for 3 hours. The product underwent Soxhlet extraction with methanol for 24 hours and was finally vacuum-dried at RT.

1.1.8 Liberation of polymers from Fe-BTC

To study the oligomeric PDA chains modified with thiols, Fe-BTC/PDA, Fe-BTC/PDA-DIP, and Fe-BTC/PDA-TDIET samples were sonicated in a 0.5 M Na2EDTA solution. After one hour, the solution turned yellow and a dark powder remained dispersed in the solution. The mixtures were kept shaking for another 12 hours and underwent subsequent washing with EDTA and then 0.01 M HCl to remove any residue Fe from the polymer. A reference sample of bare Fe-BTC was completely dissolved after one hour without any visible solid remaining in the mixture. Notably, the precipitate from PDA samples modified with thiols was much less dispersible in water compared to the precipitate from Fe-BTC/PDA (see Figure S3)

1.2 Adsorption experiments

For a typical batch adsorption experiment, 5 mg of dry MOF powder was weighed out in a 20 ml scintillation vial followed by adding 10 ml of precious metal solution. The vials were closed and kept shaking at 250 rpm on a Thermoshaker at 28 °C for 24 hours. Afterward, the solids were filtered using syringes equipped with 0.22 um PTFE filters. From the filtered solutions, 4 ml were added to a fresh centrifuge tube, and 500 µL of 37 wt.% HCl was added. The metal concentrations of the acidified samples were quantified using ICP-OES. The adsorption capacities Q_e (mg/g) were calculated based on the different metal concentrations before and after exposure to MOF-based adsorbents:

$$Q_e = \frac{(c_0 - c_e) * V}{M} \tag{1}$$

Where c_0 is the initial concentration (mg/l), c_e is the equilibrium concentration, V is the volume of the Pt solution (l) and M is the mass of the adsorbent (g). For **kinetic experiments**, 100 mg of adsorbent was added to a 250 ml capped bottle equipped with a magnetic stir bar. At time $t = 0min$, 200 ml of an 85 ppm Pt(IV) solution was added. 3 ml aliquots were taken at different time points, filtered using a 0.22 µm hydrophilic PTFE syringe filter, and added to a 15 ml centrifugation tube, acidified with 375 μl of 37 % HCl. The as-prepared samples were measured in ICP-OES for quantifying the Pt concentrations. **Mulitelement selectivity experiments** were done by preparing a solution containing 500 ppm Cu(II), 100 ppm Ni(II), 100 ppm Zn(II), and 10 ppm of Pt(IV), Au(III), and Pd(II). Adsorption experiments were done with an exposure time of 4 hours and an adsorbent loading of 1 mg/ml. The distribution constant K_d with unit ml/g was calculated as following:

$$K_d = \frac{(c_0 - c_e)}{c_e} * \frac{V}{m} \tag{2}$$

Where c_0 is the initial concentration (mg/l), c_e is the equilibrium concentration, V is the volume of the solution (l) and M is the mass of the adsorbent (g). The selectivity factor α was calculated as following:

$$\alpha = \frac{K_d^{Pt}}{K_d^{I}} \tag{3}$$

Where K_d^{Pt} is the distribution constant for Pt(IV) and K_d^{I} is the distribution constant calculated for the interfering ion Ni, Cu, and Zn, respectively.

Regeneration experiments were done in a 100 ppm Pt(IV) solution with an adsorbent loading of 4.2 mg/ml and an adsorption time of 1 hour. Desorption was done using a 0.8 M thiourea solution in 0.5 M HCl. After adsorption, the Pt-loaded powder was recovered with centrifugation and exposed to 20 ml of the leaching solution for 3 minutes, followed

by centrifugation and re-exposure to another 20 ml. Lastly, the powder was washed with 20 ml of water. The Pt desorption was calculated from the Pt concentration in each 20-ml supernatant. Three cycles were conducted to test the cyclability of the material.

1.3 Characterization

Powder X-ray diffraction **(PXRD)** patterns were collected with a Bruker D8 Discover system with a Cu K_α source (1.54056 Å) at 40 kV and 40 mA. The scanning range was 1-40 $°2\theta$ at a scanning rate of 0.5 steps per second. **Nitrogen adsorption isotherms** were measured on a Belsorp Max-II instrument at 77 K. The samples were activated at 125 °C on a separate activation station and backfilled with argon prior to measurement. X-ray photoelectron spectroscopy **(XPS)** measurements were carried out on a Kratos AXIS Supra instrument with an Al $K\alpha$ X-ray source. Data fitting was done using CasaXPS. Pt 4f doublets were fitted with a splitting of 3.35 eV and an area ratio of 0.75 was used. Analogously, for S 2p doublets, a splitting of 1.16 eV and an area ratio of 0.511 was used. Thermogravimetric analysis **(TGA)** was performed with a TA Q-Series Q500 instrument. The thermal stability profile was measured in 10 ml/min airflow and a ramp of 5 °C per minute up to 800 °C. In order to reduce the influence of different solvent content between samples, an isotherm at 125 °C for 2 hours was done and each profile was normalized to the weight after the isotherm. **FT-IR** spectra were measured with a Perking-Elmer Frontier spectrometer between 4000 and 400 cm^{-1} with a resolution of 2 cm^{-1} and a data interval of 1 cm^{-1}. **Raman** spectroscopy was carried out on a Renishaw microscope, where the focused excitation light is collected in a backscattering configuration. The powder sample is loaded onto a translational stage of a Leica microscope equipped with a 50x objective, and the excitation is a laser diode of 633 nm at 1% power to prevent degradation of the sample. The spectra were collected from 0 cm-1 to 1200 cm^{-1} with a 25 seconds acquisition time, the spectra were accumulated. Metal concentrations in aqueous solutions were determined by Agilent 5110 Synchronous Vertical Duel View **ICP-OES**. **Mass spectrum measurements** were performed on a LTQ Orbitrap FTMS instrument (Thermo Scientific) coupled with a robotic chip-based nano-ESI source. Scanning electron microscopy **(SEM)** images were acquired with a Thermo Scientific Teneo SEM instrument equipped with an in-column (Trinity) detector. Images were acquired at an accelerating voltage of 2.5 kV using a beam current of 13 pA. Energy dispersive X-ray spectroscopy **(EDX)** was conducted on a Thermo Scientific Titan Themis 60-300 in scanning transmission electron microscopy mode (**STEM**) at an accelerating voltage of 200 kV. Images were acquired using a high-angle annular dark-field (HAADF-STEM) detector with 23–200 mrad collection angle. STEM-EDX elemental maps were acquired using a beam current of 250 pA. In order to image cross sections of the MOF/polymer composites, the particles were embedded in an epoxy resin and serially sectioned in approximately 80

nm thick slices using an ultramicrotome.

1.4 XANES and EXAFS measurements

Samples for ex-situ measurements were prepared by adding 20 ml of 10 ppm, 100 ppm, and 200 ppm Pt(IV) to 20 mg of adsorbent. The mixture was kept shaking at 250 rpm for 2 hours, the powder was collected by centrifugation and vacuum dried. The powders were loaded into 1 mm glass capillaries and densely packed using cotton and finally flame sealed. Reference (bare Fe-BTC and Pt-free samples) were packed into capillaries analogously. Spectra were collected from 11.4 keV to 12.6 keV with a step size of 0.7 eV and 100 ms per data point in transmission and fluorescence mode. Each sample was scanned 10 times and averaged for data analysis. Standards from pure platinum salts ($PtCl_4$ and K_2PtCl_4) were prepared by diluting the salts with cellulose and pressing the powders into pellets for measurements. For in-situ Pt adsorption experiments, Kapton tubes with an inner diameter of 2 mm (both ends open, total length 10 cm) were filled with dry adsorbents and packed with glass wool. The length of the packed bed was approximately 1.5 cm. Plastic tubes were connected to both ends and the connections were fixed with a UV-resin. The filled Kapton tubes were placed in the Synchrotron beamline and using a peristaltic pump, a 100 ppm $PtCl_4$ (pH 3) solution was pumped through the fixed adsorption bed (flow rate = 0.5 ml/min) while measuring the Pt adsorption edge. Data were collected with a step size of 0.5 eV and 100 ms per data point in fluorescence and transmission mode. Each scan required 3 minutes and 41 seconds for acquisition.

2 Supplementary Figures and Tables

2.1 Material characterization

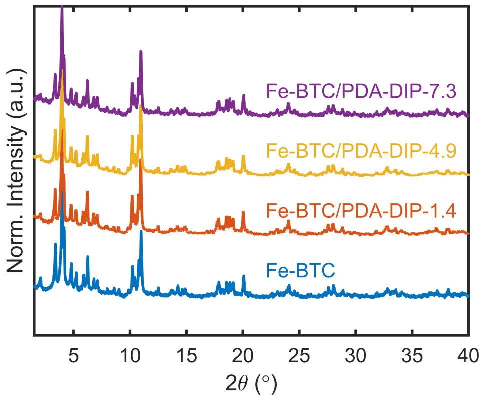

Figure S1: PXRD patterns of Fe-BTC/PDA-DIP with different loadings of DIP. Note that for completion, the powder patterns of Fe-BTC, and Fe-BTC/PDA-DIP-7.3 that are shown in the main draft are included again here.

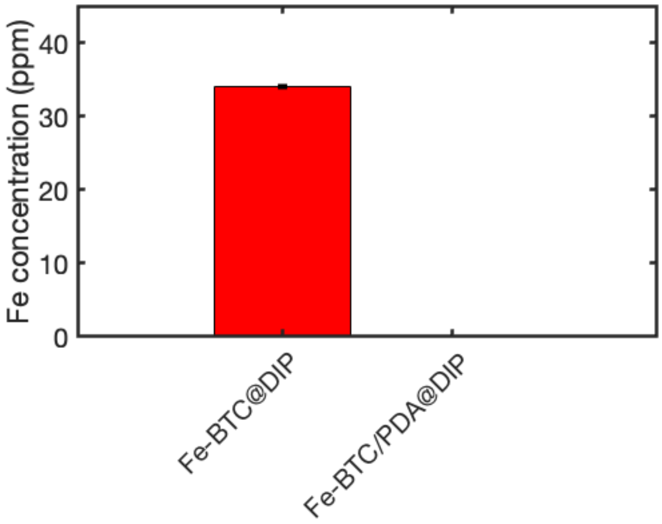

Figure S2: Measured Fe concentrations of methanol supernatant after modification of Fe-BTC/PDA with DIP and control experiments with bare Fe-BTC (no PDA). The Supernatant was collected by centrifugation followed by filtration through a 0.22 um PTFE filter.

Table S1: Elemental analysis results for post-synthetically modified Fe-BTC/PDA composites. The weight percentages of PDA and DIP were calculated using the subtotal mass of N and S, respectively, of the molecular formula of dopamine and DIP.

Sample	C%	N%	S%	PDA wt.%	DIP wt. %	DA/DIP Ratio)	S_{BET} (m^2/g)
Fe-BTC	34.83	0	–	–	–	–	1550
Fe-BTC/PDA	37.87	1.11	–	12.14	–	–	1277
Fe-BTC/PDA-DIP-1.4	38.47	1.20	0.74	13.13	1.41	1:0.15	1260
Fe-BTC/PDA-DIP-4.9	37.51	1.15	2.55	12.58	4.94	1:0.55	1006
Fe-BTC/PDA-DIP-7.3	37.74	1.12	3.78	12.25	7.32	1:0.84	1097

The PDA and DIP weight percents were calculated as following:

$$PDA\,wt.\% = N\,wt.\% * \frac{MW(dopamine)}{MW(N)} \tag{4}$$

$$DIP\,wt.\% = S\,wt.\% * \frac{MW(DIP)}{MW(S)} \tag{5}$$

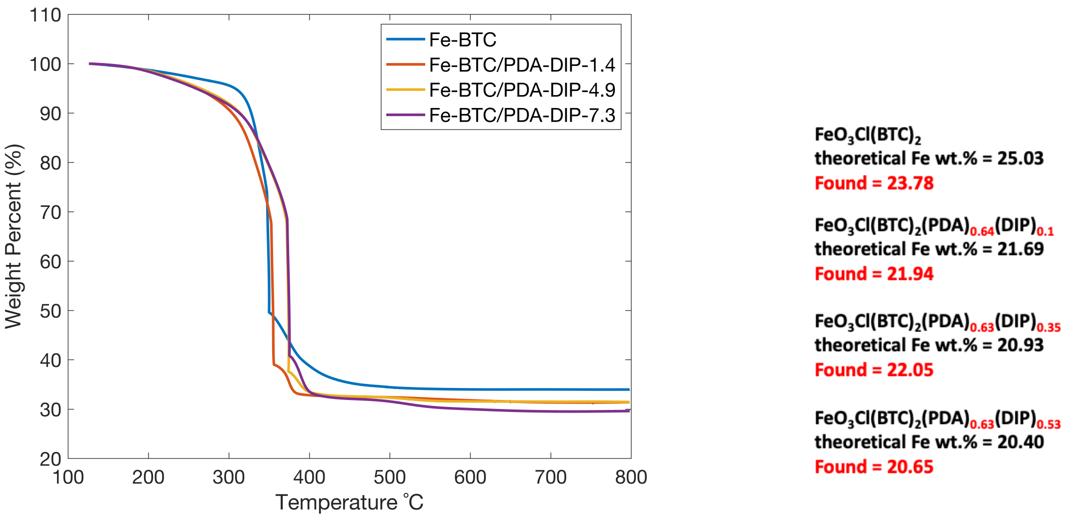

Figure S3: TGA profiles with different DIP loadings. The calculated molecular formula is shown on the right side of the graph, comparing the theoretical Fe wt. %, based on the MF, with experimental Fe wt. %. In order to exclude any influence of varying degree of solvation, the TGA profiles were normalized to the weight measured at 125 ℃, equal to the activation temperature used for N2 adsorption experiments.

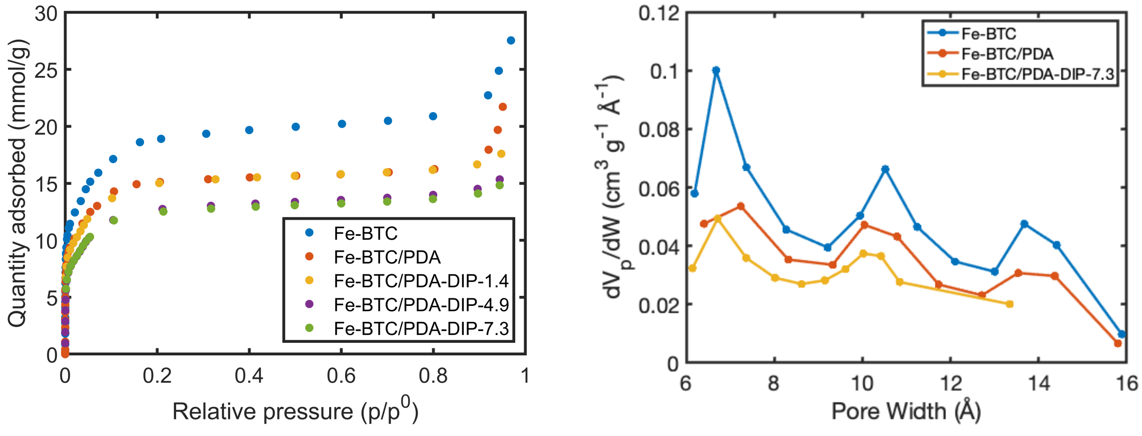

(a) **(b)**

Figure S4: (a) N_2 adsorption isotherms measured at 77 K. (b): Horvath-Kawazoe (HK) pores size distribution obtained for the bare MOF Fe-BTC and MOF/Polymer composites with different polymer loadings. As can be seen, the introduction of PDA and the following post-synthetic modification leads to a subsequentially decreased pore volume as a results form the pore filling.

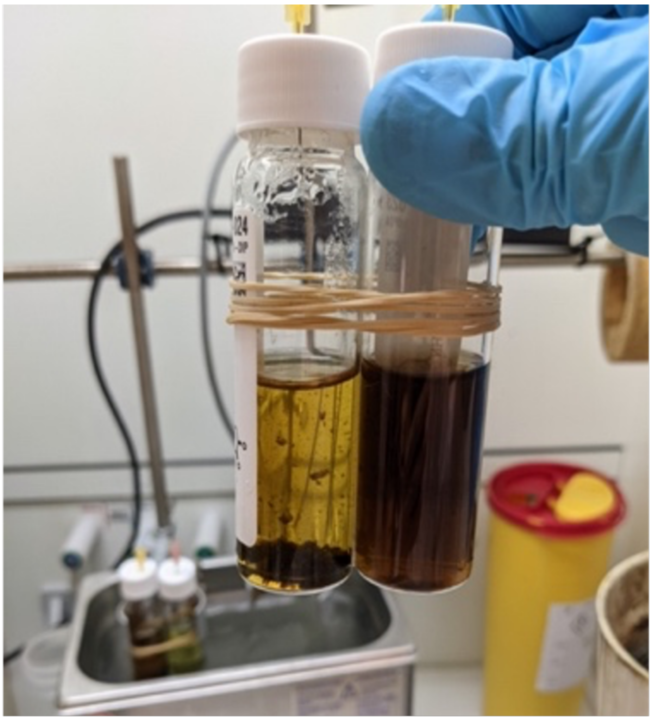

Figure S5: Digital photograph of Fe-BTC/PDA-DIP (left) and Fe-BTC/PDA (right) digested in EDTA solution.

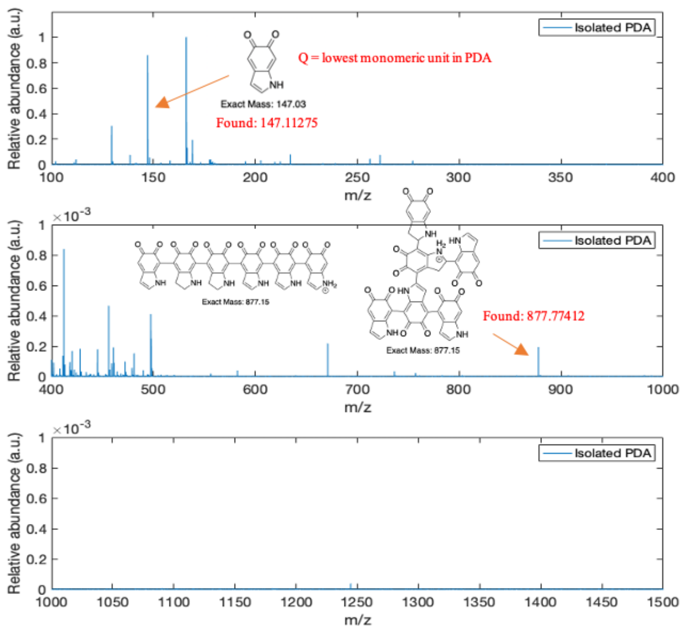

Figure S6: ESI-MS spectra of isolated PDA from Fe-BTC/PDA with EDTA and HCl. The figures are all from the same spectra, but zoomed in in different regions. Note the different scales on the y-axis.

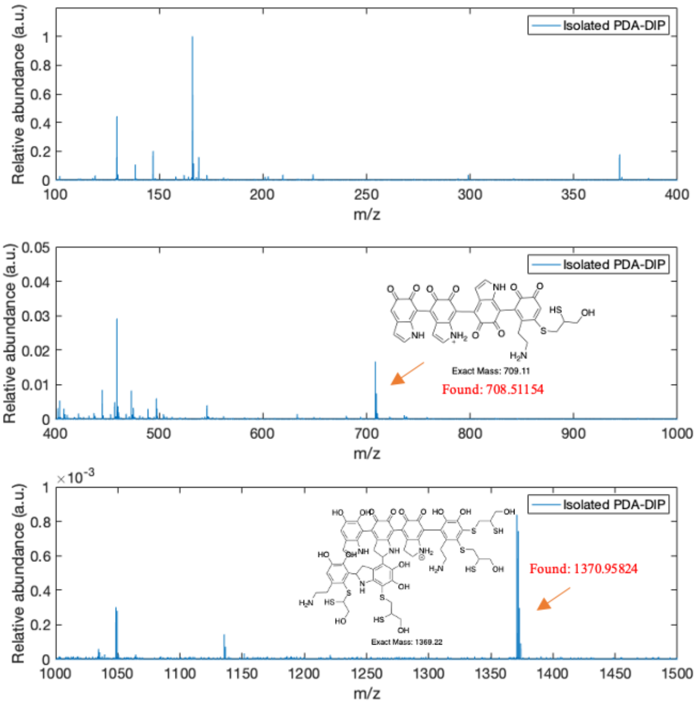

Figure S7: ESI-MS spectra of isolated PDA from Fe-BTC/PDA-DIP-7.3 with EDTA and HCl. The figures are all from the same spectra, but zoomed in in different regions. Note the different scales on the y-axis.

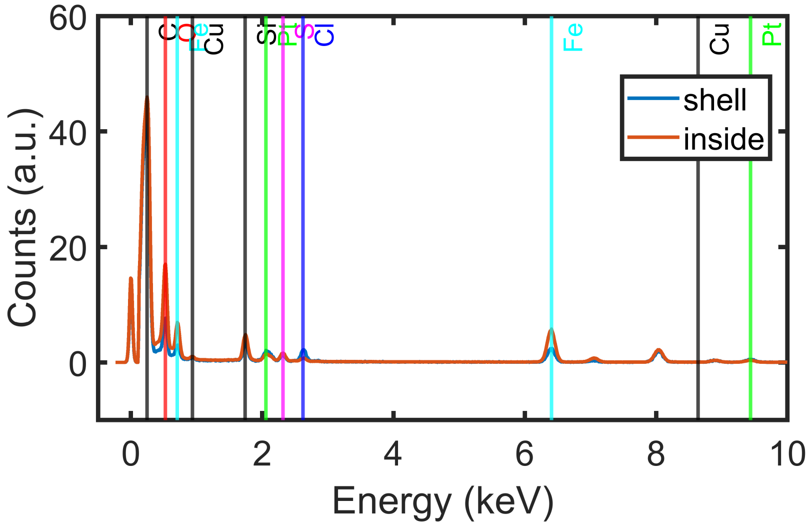

Figure S8: EDX spectra of the cross-section of Fe-BTC/PDA-DIP-7.3 soaked in a 200 ppm Pt solution.

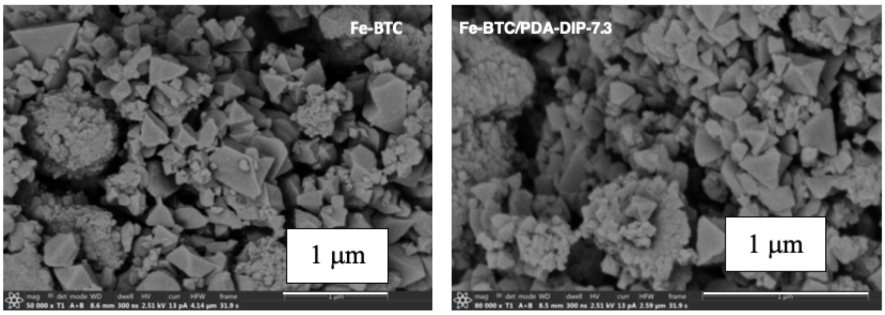

Figure S9: Scanning electron microscopy images of pristine Fe-BTC and Fe-BTC/Polymer composites.

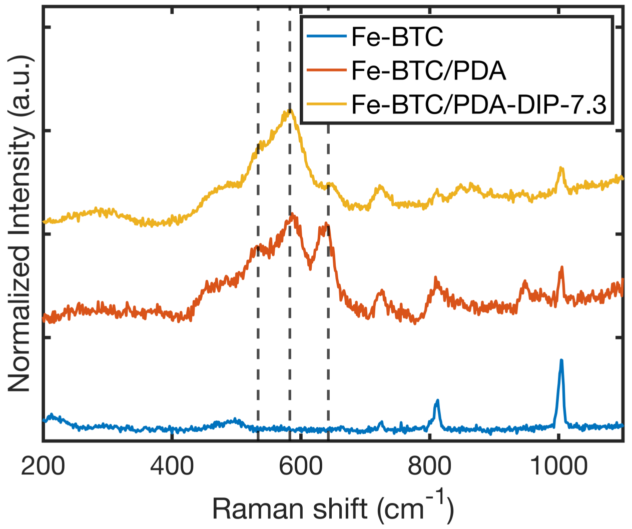

Figure S10: RAMAN spectra of MOF polymer composites before and after modification with DIP, including pristine Fe-BTC as a reference.

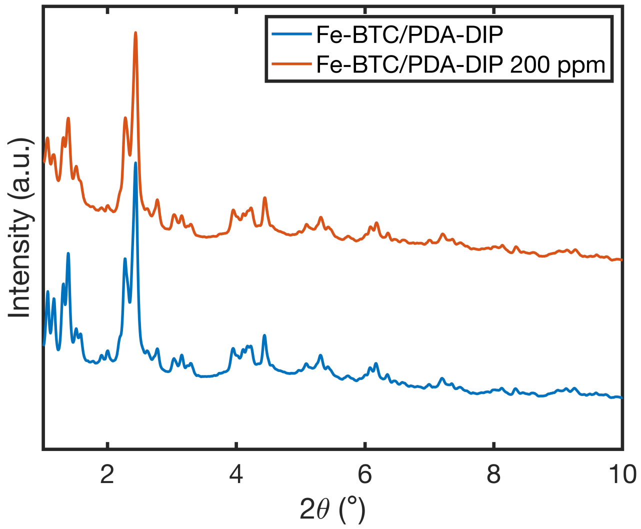

Figure S11: SPXRD pattern of the composite Fe-BTC/PDA-DIP before and after Pt adsorption.

2.2 Adsorption mechanism

The isotherm data was fitted to a Freundlich model (equation 6) using the *non-linear least squares* regression model using Matlab's *Curve Fitting Toolbox*.

$$Q_e = K_F * C_e^{1/n} \tag{6}$$

where Q_e is the experimentally determined adsorption capacity at the equilibrium concentration C_e, and K_F and $1/n$ are the fitting parameters. The fitted parameters including the corresponding R^2 value of the model are listed in the table below. The equilibrium uptake Q_{e_20} at $C_e = 20$ was taken to compare the uptake at low concentrations between the different materials. The values are given with an 80 % confidence level. The critical value for $\alpha = 0.2$ was calculated using the tinv function in Matlab's *Statistics and Machine Learning Toolbox*. the upper and lower bounds are calculated as follows:

$$lower/upper\ bound = Q_{e\_20} \pm (critical\ value * SE) \tag{7}$$

Where SE is the standard error calculated as below using the residual sum of squares (RSS) and the degree of freedom N:

$$RSS = \sum(Q_{e\_fit} - Q_{e\_exp})^2 \tag{8}$$

$$N = (number\ of\ data\ points) - 2 \tag{9}$$

$$SE = \sqrt{\frac{RSS}{N}} \tag{10}$$

Table S2: Fitting parameter of the Freundlich adsorption model in Figure 4.

Sample	K_F	$1/n$	R^2
Fe-BTC	21.909418	2.16241464	0.9712
Fe-BTC/PDA	15.024247	1.687123	0.9655
Fe-BTC/PDA-DIP-1.4	40.068564	2.231914	0.9743
Fe-BTC/PDA-DIP-4.9	80.933373	2.896829	0.9744
Fe-BTC/PDA-DIP-7.3	121.532118	3.541591	0.9815

Table S3: Data from Pt adsorption experiment with Fe-BTC.

Sample	Ce (ppm)	Ce error	Q_e (mg/g)	Qe error
Fe-BTC	55.44	8.54	102.93	1.66
Fe-BTC	82.97	14.33	211.57	22.54
Fe-BTC	176.77	4.67	233.73	0.02
Fe-BTC	383.76	3.24	343.97	6.47
Fe-BTC	559.20	4.97	406.77	21.49

Table S4: Data from Pt adsorption experiment with Fe-BTC/PDA.

Sample	Ce (ppm)	Ce error	Q_e (mg/g)	Qe error
Fe-BTC/PDA	50.34	0.56	116.35	3.45
Fe-BTC/PDA	53.65	0.08	195.86	1.71
Fe-BTC/PDA	72.73	0.87	219.65	3.74
Fe-BTC/PDA	151.52	2.82	288.90	0.14
Fe-BTC/PDA	341.46	11.13	419.89	13.58
Fe-BTC/PDA	464.97	0.73	612.98	4.71

Table S5: Data from Pt adsorption experiment with Fe-BTC/PDA-DIP-1.4.

Sample	Ce (ppm)	Ce error	Q_e (mg/g)	Qe error
Fe-BTC/PDA-DIP-1.4	31.49	0.09	129.06	0.18
Fe-BTC/PDA-DIP-1.4	34.95	0.02	233.77	4.53
Fe-BTC/PDA-DIP-1.4	53.15	0.05	278.14	2.97
Fe-BTC/PDA-DIP-1.4	129.87	2.80	329.08	2.48
Fe-BTC/PDA-DIP-1.4	299.45	3.44	523.13	3.67
Fe-BTC/PDA-DIP-1.4	459.75	11.25	623.76	29.03

Table S6: Data from Pt adsorption experiment with Fe-BTC/PDA-DIP-4.9.

Sample	Ce (ppm)	Ce error	Q_e (mg/g)	Qe error
Fe-BTC/PDA-DIP-4.9	13.74	0.30	157.69	0.56
Fe-BTC/PDA-DIP-4.9	16.40	0.10	261.83	2.63
Fe-BTC/PDA-DIP-4.9	34.93	0.37	309.55	8.64
Fe-BTC/PDA-DIP-4.9	109.15	2.45	366.74	1.99
Fe-BTC/PDA-DIP-4.9	281.80	6.01	548.28	9.91
Fe-BTC/PDA-DIP-4.9	433.33	5.37	683.74	10.95

Table S7: Data from Pt adsorption experiment with Fe-BTC/PDA-DIP-7.3.

Sample	Ce (ppm)	Ce error	Q_e (mg/g)	Qe error
Fe-BTC/PDA-DIP-7.3	6.84	0.12	179.18	1.53
Fe-BTC/PDA-DIP-7.3	11.57	0.10	281.39	2.93
Fe-BTC/PDA-DIP-7.3	25.45	0.28	331.80	0.58
Fe-BTC/PDA-DIP-7.3	99.68	3.68	396.29	3.64
Fe-BTC/PDA-DIP-7.3	264.05	3.75	583.40	7.49
Fe-BTC/PDA-DIP-7.3	412.95	7.12	684.01	26.84

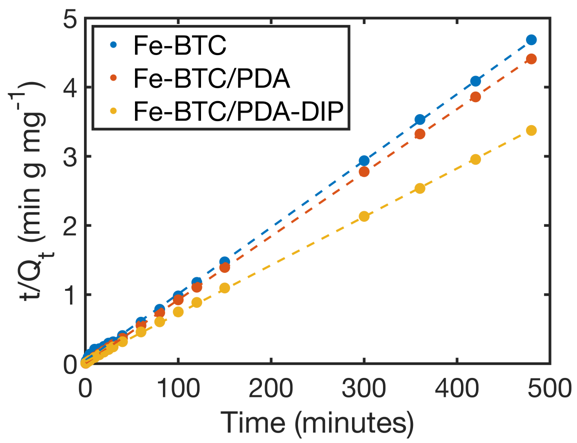

Figure S12: adsorption data (solid dots) fitted to a pseudo-second-order kinetic model (dotted line).

The adsorption data was fitted to the linearized form of the pseudo-second-order kinetic model (equation 11) using with a simple linear regression model using the *mldivide* operator in Matlab:

$$\frac{t}{Q_t} = \frac{1}{k_2 Q_e^2} + \frac{t}{Q_e} \tag{11}$$

where t is the time in minutes, Q_t is the platinum uptake at time t in mg/g, Q_e is the equilibrium uptake in mg/g, and k_2 is the rate constant in g*mg^{-1}min^{-1}. The data were fitted with a linear regression using a least square fit. Q_e was obtained from the slope and k_2 is obtained from the y-intercept. Table S8 shows the calculated parameters for the three adsorption experiments.

Table S8: The parameters k_2 and Q_e were obtained from the pseudo-second-order kinetic model (equation 11) and calculated as described in the section above. The initial concentration C_0 was 75 ppm Pt and the adsorbent dosage was 0.5 mg/ml. The error of the rate constant k_2 was determined by calculating standard deviation of the rate constants from the triplicate data-set.

Sample	k_2 (g mg^{-1} min^{-1})	$Q_{e,pred}$	$Q_{e,exp}$	$Q_{e,err}$ (%)	R^2	MSE	χ^2
Fe-BTC	0.0020079 $\pm$ 5.89*10^{-5}	104.0341	102.224	0.9614	0.9997	7.8554*10^{-4}	0.0134
Fe-BTC/PDA	0.0117768 $\pm$ 1.4*10^{-2}	108.9086	108.884	0.1193	1.0000	5.5438*10^{-5}	9.4244*10^{-4}
Fe-BTC/PDA-DIP-7.3	0.0017365 $\pm$ 2.57*10^{-5}	143.1735	142.224	0.5669	0.9998	2.1899*10^{-4}	0.0037

Wheres $Q_{e,err}$, R^2, and χ^2 are calculated as followed:

$$Q_{e,err} = \frac{Q_{e,exp} - Q_{e,pred}}{Q_{e,exp}} * 100 \tag{12}$$

$$R^2 = 1 - \frac{((t/Q_t)_{exp} - (t/Q_t)_{pred})^2}{(t/Q_t)_e xp - mean(t/Q_t)_e xp)} \tag{13}$$

$$MSE = \sum \frac{(t/Q_t)_{exp} - (t/Q_t)_{pred})^2}{N-2} \tag{14}$$

$$\chi^2 = MSE * (N-2) \tag{15}$$

Where N is the number of experimental data points and 2 is the number of parameters in the PSO model. $Q_{e,exp}$ $Q_{e,pred}$ are the experimental and predicted capacities, respectively.

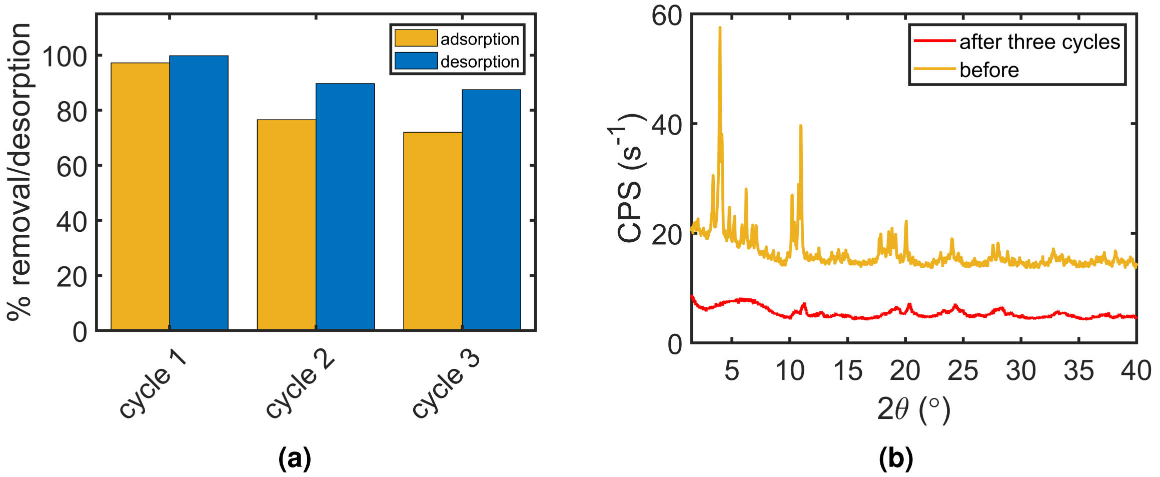

Figure S13: a) Regeneration experiment. Pt desorption was done using a thiourea (0.8 M) in aqueous 0.5 M HCl solution. The drop in removal efficiency after the first cycle is attributed to limited stability of the MOF in the leaching solution. In the first cycle, a significant amount of iron leaching was detected. Adsorption conditions: 4.2 mg/ml adsorbent dosage, Pt concentration = 10 ppm, adsorption time: 1 hour. Desorption condition: 2 times soaking in 20 ml leaching solutions for 3 minutes followed by centrifugation, followed by a washing step with MQ water and vacuum drying of the powder for the next cycle. The desorption percentage is calculated from the sum of the Pt concentrations in the three supernatant solutions. b) shows the PXRD pattern after three cycles. Clearly, the material becomes more amorphous after repeated adsorption-desorption cycles.

Table S9: Comparison of material performance across the literature.

Material	C_0 (mg/L)	C_e (mg/L)	Q_e (mg/g)	K_d (ml/g)	ads. dosage (mg/ml)	Matrix	Ref.
Zr-BDC-NH2	1600	1503[a]	193	129	0.5	Pt(IV)	Lin *et al.* [1]
Zr-BDC-NH2	100	50[a]	100	2'000	0.5	Pt(IV)	Lin *et al.* [1]
Cr-BDC-NH2	100	82.5[a]	35.1	425	0.5	Pt(IV)	Lim *et al.* [2]
Fe-BTC/PDA-DIP-7.3	75	5.7	142	24'912	0.5	Pt(IV)	This work
Fe-BTC/PDA-DIP-7.3	9.26	0.15	9.15	62'845	1.0	Pt, Cu, Ni, Zn, Pd, Au, Pb	This work
Amberjet 4200	125	25[a]	100	3000	1.0	Pt(IV)	Mao *et al.* [3]
PAA/HCl-modified *E. coli*	125	5.7[a]	119.3	20'930	1.0	Pt(IV)	Mao *et al.* [3]

[a] calculated based on the reported Q_e and C_e values.

Table S10: The binding energies of the synthetic peaks used in the fitting in Figure 4A. The FWHM were fixed for each model, and the values are given in the last column.

	Pt (II) / eV		Pt (IV) / eV		
Fe-BTC/PDA-DIP-7.3	$\{4f_{7/2}\}$	$\{4f_{5/2}\}$	$\{4f_{7/2}\}$	$\{4f_{5/2}\}$	FWHM
10 ppm Pt(IV)	72.76	76.11	74.70	78.05	1.42
100 ppm Pt(IV)	72.69	76.04	74.88	78.23	1.40
200 ppm Pt(IV)	72.83	76.18	75.05	78.40	1.49

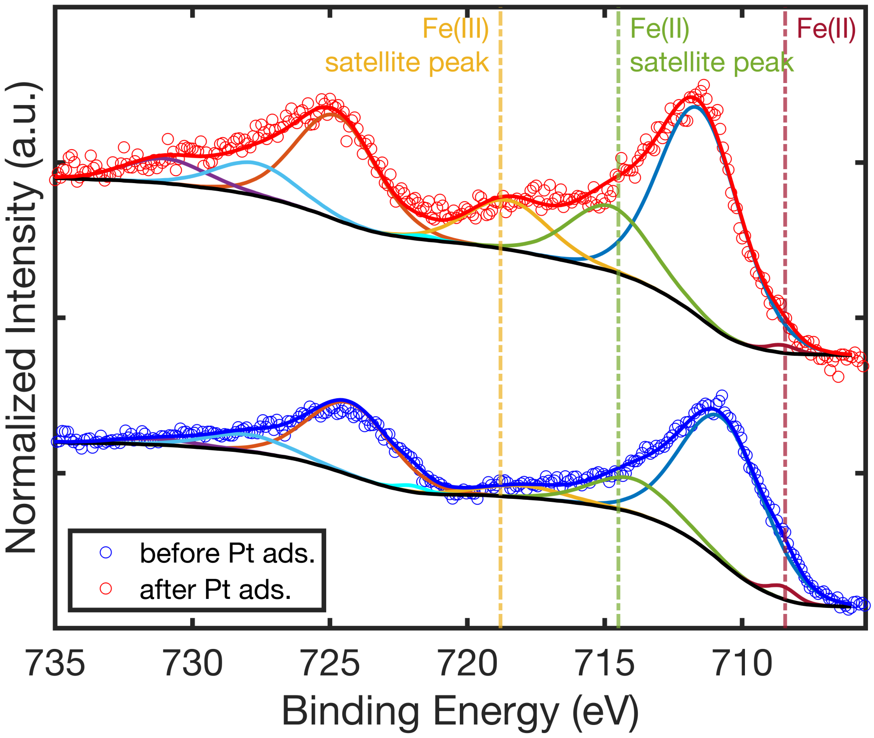

Figure S14: High resolution XPS Fe 2p region of Fe-BTC/PDA-DIP-7.3. We see that the small contribution from Fe(II) disappears after soaking the sample in a 10 ppm Pt(IV) solution, while the distinct Fe(III) satellite peak becomes more pronounced. More details regarding the exact binding energies of fitted peaks can be found in the table below.

Table S11: The binding energies of the synthetic peaks used in the fitting in Figure S24. A peak splitting of 13.1 eV was used for the initial fit but was relaxed for reducing the STD. Each peak position was constrained within a 1 eV range. Yamashita and Hayes was consulted for the fitting. [4]

	Fe $2p_{3/2}$	$Fe_{3/2}$ satellite	Fe $2p_{1/2}$	$Fe_{1/2}$ satellite
Before ads.				
Fe^{3+}	710.70	718.00	724.27	731.34
Fe^{2+}	708.51	714.00	721.92	728.00
After ads.				
Fe^{3+}	711.52	718.53	724.75	731.00
Fe^{2+}	708.52	714.80	721.62	727.69

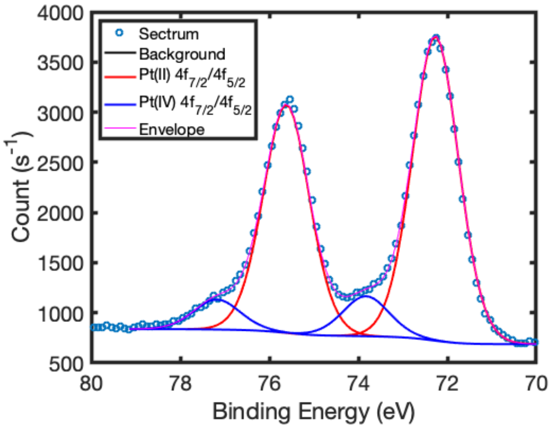

(a) Pt 4f region of DIP-Pt complex

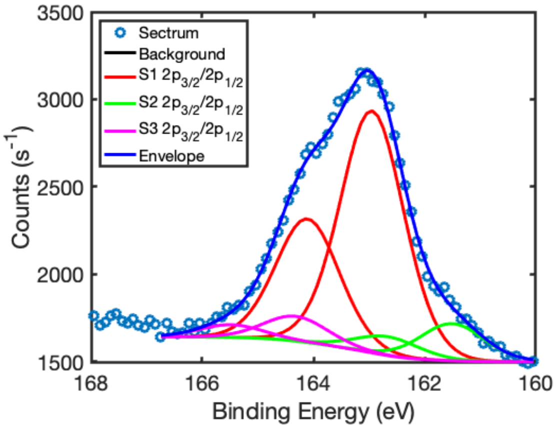

(b) S 2p region of DIP-Pt region

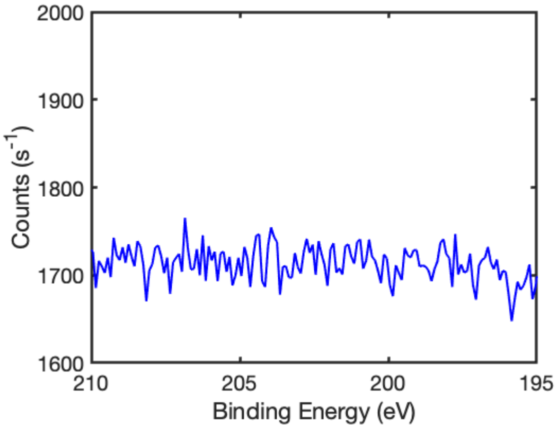

(c) Cl 2p region of DIP-Pt region

Figure S15: X-ray photoelectron spectra of the (a) Pt 4f region, (b) S 2p and (c) Cl 2p region of the DIP-Pt precipitate prepared as described in the experimental method sections.

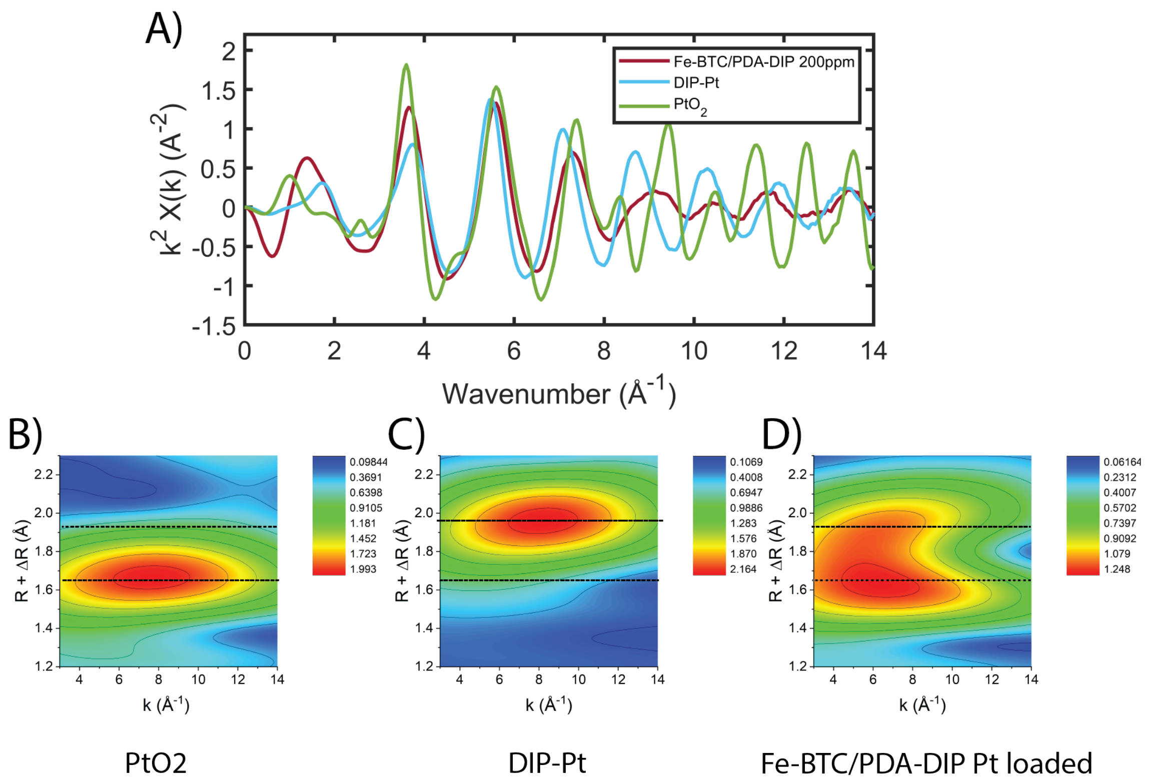

Figure S16: A) K-space plot comparing Pt loaded Fe-BTC/PDA-DIP with PtO_2 and DIP-Pt. B-D) Shows the wavelet transform (WT-EXAFS) where the k-R deconvolutions highlight that the Pt-soaked sample contains contributions from a Pt-O and a Pt-S bond. Wavelet transforms extend Fourier transforms (applied onto EXAFS data-sets) is a technique that efficiently separates contributions of a waveform into both k and R-space. More information can be found here: Munoz *et al.*, [5] and Funke *et al.* [6]

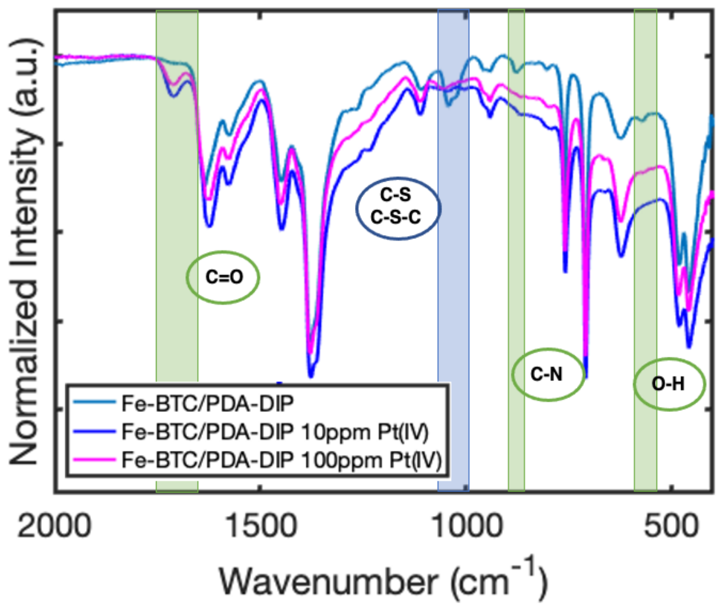

Figure S17: FT-IR spectra of Fe-BTC/PDA-DIP-7.3 before and after soaking in platinum solution.

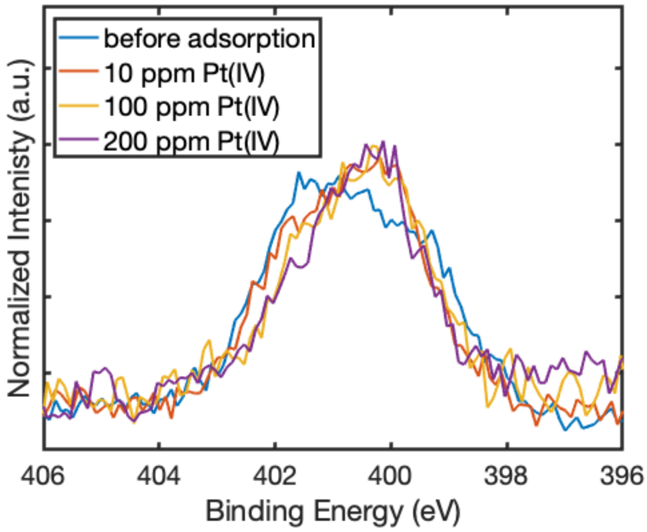

Figure S18: XPS of the N 1s region of Fe-BTC/PDA-DIP-7.3 before and after soaking in platinum solutions with increasing concentration.

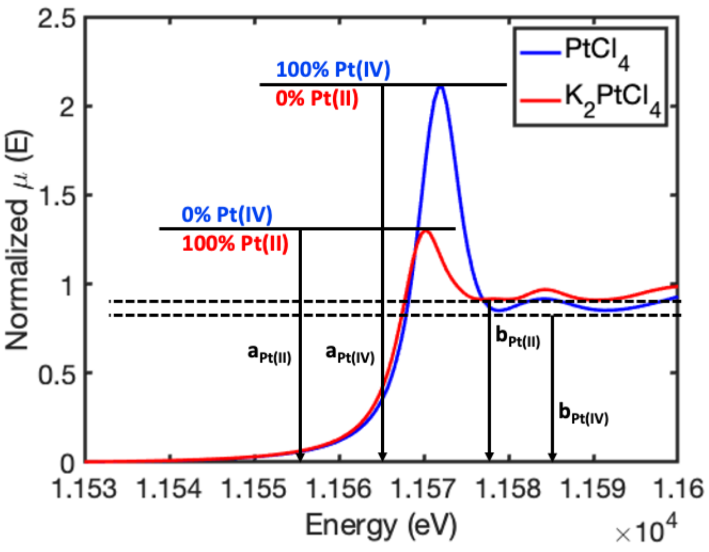

Figure S19: (a) example of how the white line intensity a and the post-edge minima b in XANES are defined.)

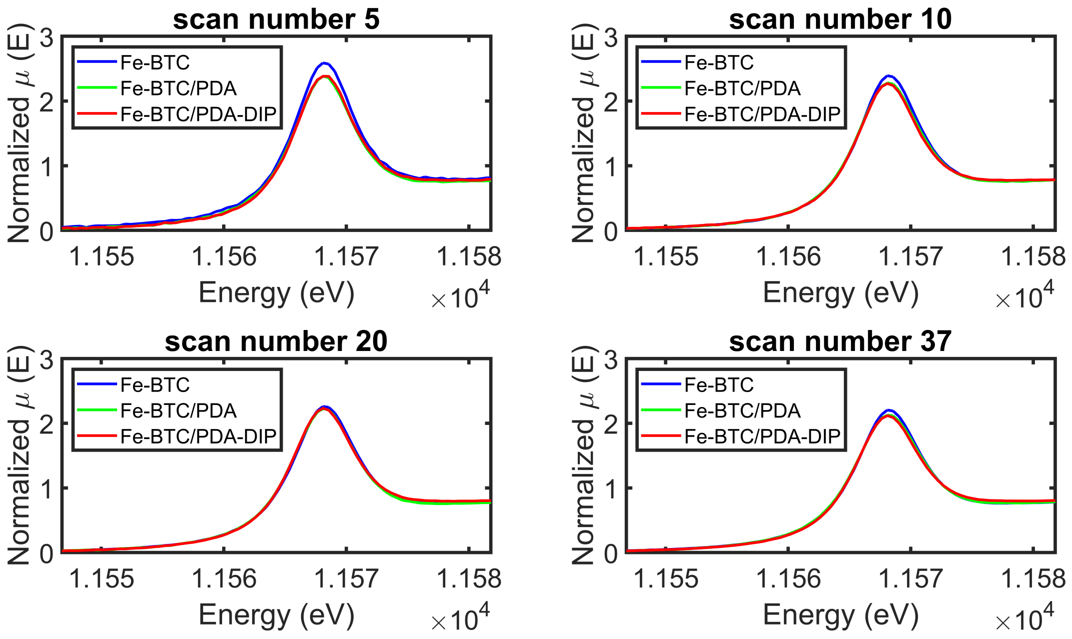

Figure S20: Normalized transmission XANES of Fe-BTC, Fe-BTC/PDA and Fe-BTC/PDA-DIP-7.3 of different time points during the in-situ platinum adsorption experiments. The (a/b) ratios were calculated from these XANES and used to generate Figure 6A in the main draft.

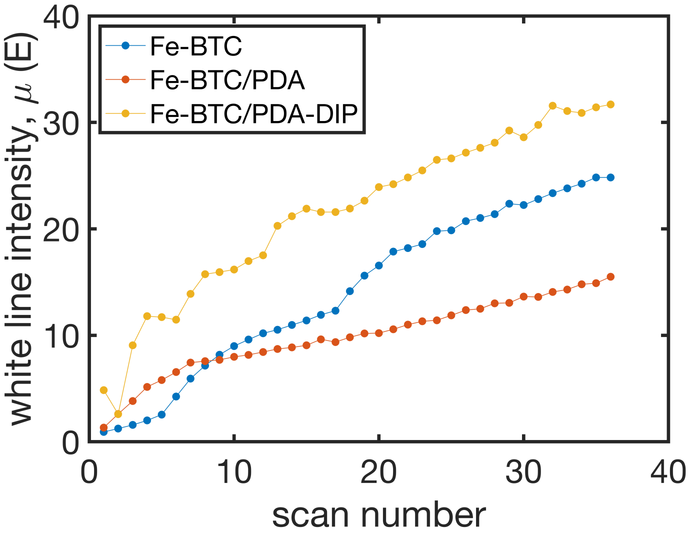

Figure S21: The figure shows the peak maxima of the white line intensity obtained from the in-situ XANES as a function of scan number. The increases intensity of non-normalized XANES indicates a higher concentration of platinum. By comparing different samples, we see that platinum is accumulated faster in Fe-BTC/PDA-DIP-7.3 compared to the bare MOF and Fe-BTC/PDA.

2.3 Extension of the post-synthetic modification strategy

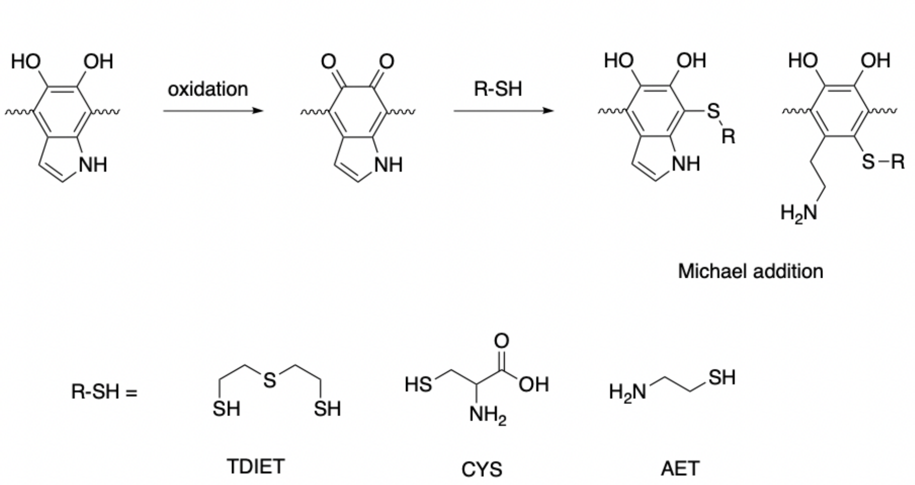

Scheme S1: Reaction scheme of post-synthetic modification of PDA with different thiols, including 2,2-thiodiethanethiol (TDIET), cysteine (CYS), and 2-aminoethanethiol (AET)(order from left to right).

Table S12: Elemental analysis results for post-synthetically modified Fe-BTC/PDA composites using TDIET, CYS, and AET

Sample	C%	H%	N%	S%
Fe-BTC/PDA-DIP	37.74	1.12	3.78	12.25
7.32				
Fe-BTC/PDA-TDIET	35.92	2.67	0.91	13.77
Fe-BTC/PDA-CYS	35.72	2.96	3.74	7.11
Fe-BTC/PDA-AET	36.38	2.62	3.01	5.20

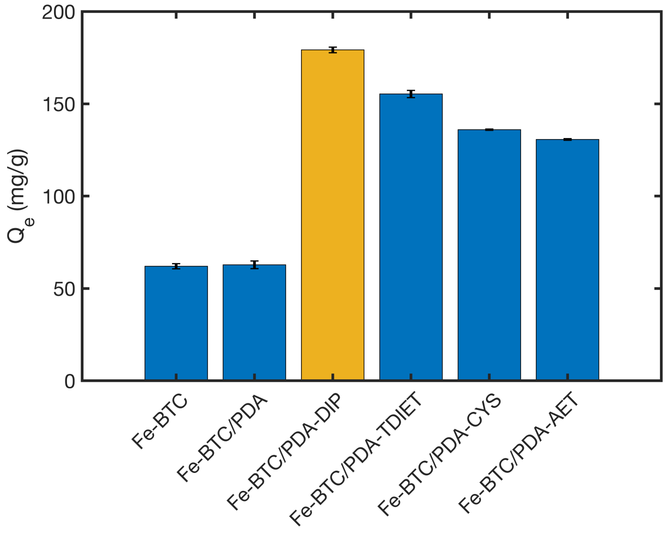

Figure S22: Pt uptake measured from a $PtCl_4$ solution (90 ppm, Pt) with an adsorption dosage of 0.5 mg/ml and an adsorption time of 24 hours.

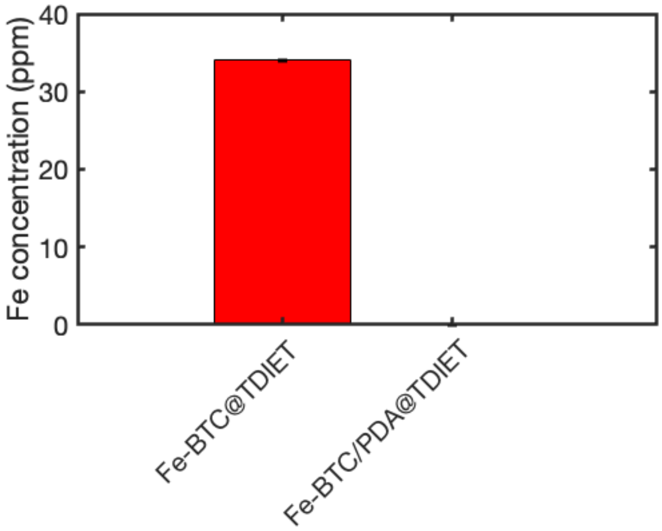

Figure S23: Fe concentration measured in the supernatant after soaking Fe-BTC and Fe-BTC/PDA to TDIET in methanol for 12 hours

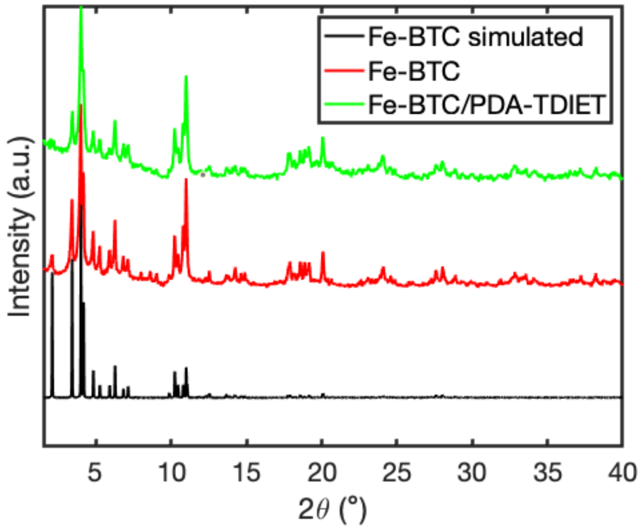

Figure S24: PXRD patterns of Fe-BTC/PDA modified with TDIET, compared with the pattern of the bare MOF and the simulated structure of Fe-BTC, showing that the crystallinity of the parent structure remains unchanged after grafting different thiols to PDA.

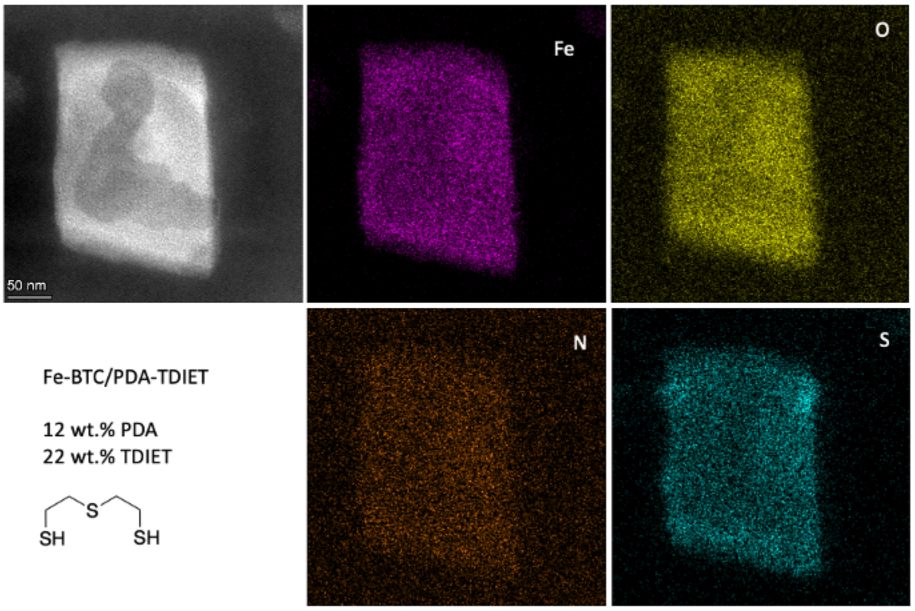

Figure S25: HAADF-STEM images of sliced single crystals of Fe-BTC/PDA-TDIET, including the EDX elemental mapping for Fe, O, N, and S of the MOF/Polymer composites.

References

[1] Shuo Lin, D Harikishore Kumar Reddy, John Kwame Bediako, Myung-Hee Song, Wei Wei, Jeong-Ae Kim, and Yeoung-Sang Yun. Effective adsorption of pd (ii), pt (iv) and au (iii) by zr (iv)-based metal–organic frameworks from strongly acidic solutions. *Journal of Materials Chemistry A*, 5(26):13557–13564, 2017.

[2] Che-Ryong Lim, Shuo Lin, and Yeoung-Sang Yun. Highly efficient and acid-resistant metal-organic frameworks of mil-101 (cr)-nh2 for pd (ii) and pt (iv) recovery from acidic solutions: Adsorption experiments, spectroscopic analyses, and theoretical computations. *Journal of hazardous materials*, 387:121689, 2020.

[3] Juan Mao, Sok Kim, Xiao Hui Wu, In-Soeb Kwak, Tao Zhou, and Yeoung-Sang Yun. A sustainable cationic chitosan/e. coli fiber biosorbent for pt (iv) removal and recovery in batch and column systems. *Separation and Purification Technology*, 143:32–39, 2015.

[4] Toru Yamashita and Peter Hayes. Analysis of xps spectra of fe2+ and fe3+ ions in oxide materials. *Applied surface science*, 254(8):2441–2449, 2008.

[5] Manuel Munoz, Pierre Argoul, and François Farges. Continuous cauchy wavelet transform analyses of exafs spectra: A qualitative approach. *American mineralogist*, 88(4):694–700, 2003.

[6] H Funke, AC Scheinost, and Marina Chukalina. Wavelet analysis of extended x-ray absorption fine structure data. *Physical Review B*, 71(9):094110, 2005.
